# Comparative and phylogenetic analysis of the complete chloroplast genomes of 10 *Artemisia selengensis* resources based on high-throughput sequencing

**DOI:** 10.1186/s12864-024-10455-3

**Published:** 2024-06-05

**Authors:** Yuhang Wang, Qingying Wei, Tianyuan Xue, Sixiao He, Jiao Fang, Changli Zeng

**Affiliations:** 1 Hubei Engineering Research Center for Protection and Utilization of Special Biological Resources in the Hanjiang River Basin, School of Life Science, Jianghan University, Jianghan University, Wuhan, Hubei China; 2 School of Medicine, Jianghan University, Wuhan, Hubei China

**Keywords:** *Artemisia selengensis*, Chloroplast genome, Genomic structure, Phylogenetic analysis, Comparative analysis, High-throughput sequencing

## Abstract

**Background:**

*Artemisia selengensis*, classified within the genus *Artemisia* of the Asteraceae family, is a perennial herb recognized for its dual utility in culinary and medicinal domains. There are few studies on the chloroplast genome of *A. selengensis*, and the phylogeographic classification is vague, which makes phylogenetic analysis and evolutionary studies very difficult.

**Results:**

The chloroplast genomes of 10 *A. selengensis* in this study were highly conserved in terms of gene content, gene order, and gene intron number. The genome lengths ranged from 151,148 to 151,257 bp and were typical of a quadripartite structure with a total GC content of approximately 37.5%. The chloroplast genomes of all species encode 133 genes, including 88 protein-coding genes, 37 tRNA genes, and 8 rRNA genes. Due to the contraction and expansion of the inverted repeats (IR), the overlap of *ycf1* and *ndhF* genes occurred at the inverted repeats B (IRB) and short single copy sequence (SSC) boundaries. According to a codon use study, the frequent base in the chloroplast genome of *A. selengensis*’ third codon position was A/T. The number of SSR repeats was 42–44, most of which were single nucleotide A/T repeats. Sequence alignment analysis of the chloroplast genome showed that variable regions were mainly distributed in single copy regions, nucleotide diversity values of 0 to 0.009 were calculated by sliding window analysis, 8 mutation hotspot regions were detected, and coding regions were more conserved than non-coding regions. Analysis of non-synonymous substitution (Ka) and synonymous substitution (Ks) revealed that *accD*, *rps12*, *petB*, and *atpF* genes were affected by positive selection and no genes were affected by neutral selection. Based on the findings of the phylogenetic analysis, *Artemisia selengensis* was sister to the genus *Artemisia Chrysanthemum* and formed a monophyletic group with other *Artemisia* genera.

**Conclusions:**

In this research, the present study systematically compared the chloroplast genomic features of *A. selengensis* and provided important information for the study of the chloroplast genome of *A. selengensis* and the evolutionary relationships among *Asteraceae* species.

**Supplementary Information:**

The online version contains supplementary material available at 10.1186/s12864-024-10455-3.

## Background

*Asteraceae* is the first family of dicotyledonous plants, currently, there are about 1000 genera and 25,000–30,000 species in the family, and there are about 200 genera and more than 2000 species in China, which are distributed all over the country [1, 2]. As the largest genus in *Asteraceae* [3], *Artemisia* has about 300 species. It is mainly found in temperate, cold-temperate, and subtropical regions of Asia, Europe, and North America. In many countries, most *Artemisia* plants are used as herbal medicines. For example, *A. annua* is used as a treatment for malaria because of its rich content of artemisinin [4]; the Dragon Boat Festival, a traditional Chinese festival, uses. *argyi* to repel insects and kill viruses. As a perennial herb of the genus *Artemisia* in the family *Asteraceae*, *A.a selengensis* has rhizomes, young stems are green or purple, young leaves are mostly light green, and old leaves are dark green. The leaves are mostly oval or lance-shaped in shape, with white tomentum on the back, and the whole plant grows upright or obliquely upward. The plant itself has a clear fragrance, the stalks are crisp and tender, rich in protein, fatty acids, and trace elements [5], with a delicious flavor and rich nutrition, and is widely grown mainly as a vegetable in China. *A. selengensis* contains various chemical substances such as flavonoids, chlorogenic acid, and reducing sugars. It is the polysaccharides, chlorogenic acid, and other bioactive components present within the plant, that have demonstrated effects in anti-tumor, antioxidant and free radical scavenging [6–8], which can improve liver function [8] and lower blood sugar [9]. It is also used in tea making, yogurt fermentation, functional shampoos, and cosmetics development [10, 11].

Chloroplasts are important organelles with independent genetic material and capable of photosynthesis, commonly found in terrestrial plants, algae, and a few protists [12, 13], showing matrilineal inheritance in most angiosperms. The chloroplast genomes are relatively conserved in structure [14] and has a typical tetrad structure of a circular genome with a genome size ranging from 120 to 160 kb [15], including a large single-copy region (LSC), a small single-copy region (SSC), and these two single copy regions are separated by two inverted repeat regions (IR), where the inverted repeat regions are pairs of repeats with equal length and opposite orientation sequences [16]. Chloroplast genomes are so commonly used in angiosperms, gymnosperms, and ferns for phylogenetic and comparative genomic investigations [17]. Chloroplast genomes are inherited either paternally or maternally and may be utilized as a legitimate barcode for species identification as well as the creation of additional possible identifying markers [18].

*Artemisia* species are diverse, complicated in genetic relationships, and have ambiguous taxonomic relationships based on morphology. As organelle genomes with highly conserved genetic information, the chloroplast genomes are widely used for genome evolution studies [19, 20]. Many researchers have used single gene data (*accD*, *ycf1*, *rbcL*, *matK*, *ndhF*, *rps11*) and IGS data (*psbA-trnH*, *trnS-trnC*, *trnS-trnfM*, *trnL-trnF*) for phylogenetic analysis of *Artemisia* [21–27], however, these chloroplast single-gene molecular markers do not work for all plant taxa and only supply limited information at the subspecies level [28]. In contrast, there is little information on the chloroplast genomes of *A. selengensis* in the current database, and it is unclear whether the chloroplast genomes of *A. selengensis* resources differ from one region to another. Therefore, in this work, chloroplast whole-genome sequencing, assembly, and annotation of 10 *A. selengensis* materials from 6 regions using second-generation sequencing technology not only enriched the existing genetic information of the chloroplast genome of *A. selengensis*, which was helpful for phylogenetic and taxonomic studies but also provided genetic information for the conservation of *A. selengensis* germplasm resources. To better understand the evolution of the chloroplast genome structure of *A. selengensis* and to clarify the evolutionary relationships between *A. selengensis* and other *Artemisia* species, genome structure analysis and comparative genomic research were also carried out.

## Methods

### Samples collection

Ten *A. selengensis* germplasm resources were collected from 6 provinces in China. The material’s number, source, and GenBank number are listed in Table 1. The labels for 10 different *A. selengensis* materials were HWB, HWS, HQ, HY, HC, JN1, JN2, AC, JS, and YN.

**Table 1:** Ten germplasm resources of *A. selengensis* from six provinces

ID	Source	GenBank numbers
HWB	Baishazhou Town, Wuhan City, Hubei Province	ON931227
HWS	Shamao Town, Wuhan City, Hubei Province	ON942235
HQ	Qichun City, Hubei Province	ON968864
HY	Yueyang City, Hunan Province	ON921081
HC	Chenzhou City, Hunan Province	ON968865
JN1	No.1 Nanjing City, Jiangsu Province	ON931228
JN2	No.2 Nanjing City, Jiangsu Province	ON960154
AC	Chuzhou City, Anhui Province	ON960153
JS	Shangrao City, Jiangxi Province	ON942234
YN	Qujing City, Yunan Province	ON968863

### Chloroplast genome sequencing

More than 0.5 g of fresh leaves were taken from each material separately, kept in discolored silica gel, and then sequenced by Illumina high-throughput sequencing platform from Beijing Novogene Biotechnology Co., Ltd. A total of 56.5 G of raw data and 56.15 G of filtered clean data were generated by sequencing. The clean data were utilized to assemble the chloroplast complete genome. The base quality values of the sequencing results were all above 97% for Q20 and above 92% for Q30 (Supplementary Table S1).

### Genome assembly and annotation

In this study, the chloroplast genomes of *A. selengensis* were assembled using GetOrganelle v1.7.5.3 software [29]. We used the published complete chloroplast genome of *A. selengensis* downloaded from NCBI [30] (GenBank accession: NC_039647) as a reference for chloroplast genome annotation of 10 *A. selengensis* materials, using CPGAVAS2 (http://www.herbalgenomics.org/cpgavas) online software and PGA software [31] to annotate the chloroplast genomes of *A. selengensis*. By using Geneious v8.0.4 software [32], we compared the number of annotated chloroplast genome genes, added missing genes manually, verified the CDS sequences rigorously, and manually modified the start codon and stop codon of the misannotated genes. If a gene was present as a shortened partial copy of another gene or had an internal stop codon in comparison to other homologous genes, it was deemed to be a pseudogene. The annotated GenBank file was converted into a five-column tab-delimited annotation file using GB2Sequin [33], and the chloroplast genome annotation files and the complete FASTA sequence files for 10 materials were submitted to GenBank via Bankit and specific accession numbers were acquired (Table 1). The annotated chloroplast genomes were visualized using the online software Chloroplot (https://irscope.shinyapps.io/Chloroplot).

### Structural characterization and comparative chloroplast genome analysis

Geneious v8.0.4 software was used to calculate the whole genome length, length of each region (large single-copy region, small single-copy region, inverted repeats), gene composition and position distribution, base composition, and GC (AT) content to analyze the characteristics of the chloroplast genomes of *A. selengensis*.

### Boundary regions and comparative analysis

Variations in gene sequences at the boundary junctions of the 4 regions are observed across different plant species. The main reason for the variation in chloroplast genome length is the expansion and contraction of the IR region. We used the CPJSdraw-boundary map drawing tool (http://cloud.genepioneer.com:9929) of the JSHYCloud Platform to analyze and compare the boundary regions between the large single-copy region (LSC) and the IR region and between the small single-copy region (SSC) and the IR region.

### Codon usage analysis

Codon usage bias (CUB) refers to the phenomenon that codons have the characteristics of degeneracy in the process of gene translation between different species or within the same species, that is, one amino acid corresponds to different codons, resulting in some codons using more than other synonymous codons [34]. CUB is a useful tool for understanding genetic and evolutionary processes, and the analysis of codon usage bias in genes can help determine these genes’ origin and evolutionary history. In this study, CodonW software was used to analyze the codon preferences and the results were visualized for graphing using R software. By employing CodonW and CUSP software, we calculated the effective number of codons (ENC), relative synonymous codon usage (RSCU), and the overall GC content (GCall) for each gene. Concurrently, the GC content at the three positions of codons was recorded, denoted as GC1, GC2, and GC3, respectively, with the GC content at the third position of synonymous codons represented as GC3s. To reduce errors, protein-coding sequences needed to be screened, requiring each CDS sequence to be a multiple of 3, ≥ 300 bp in length, each containing a start codon and a stop codon, with no stop codon inside the sequence, while duplicate sequences were removed, and finally, all 53 CDS sequences were retained for codon analysis.

### Scattered repeats sequence and SSRs analysis

Forward, reverse, complementary and palindromic repeats in the chloroplast genome of *A. selengensis* were detected using the REPuter (https://bibiserv.cebitec.uni-bielefeld.de/reputer) with parameters set to Hamming distance of 3, maximum calculated repeats of 50 and repeat size > 30 bp. Simple sequence repeats (SSR) were detected using MISA (https://webblast.ipk-gatersleben.de/misa/index.php) with nucleotide motifs of 1–6, parameters using default values, and the minimum number of repeats for single nucleotide, dinucleotide, trinucleotide, tetranucleotide, pentanucleotide, and hexanucleotide is set to 10, 6, 5, 5, 5 and 5 respectively.

### Comparative genomic analysis

Comparing chloroplast genome sequences provides a reference for discovering sequence variants and identifying mutation hotspot regions, as well as detecting gene loss and duplication events. Mutation hotspot regions obtained from chloroplast genome sequences can also be used as effective molecular markers for species identification and population genetics [35, 36]. mVISTA is an online tool for multiple DNA sequence alignment that allows sequence similarity to be assessed by comparing coding and non-coding regions, introns, and exons [37]. In this study, the whole chloroplast genomes of 10 *A. selengensis* were compared and visualized using mVISTA (http://genome.lbl.gov/vista/index.shtml). The published genome of *A. selengensis* (NC_039647) was selected as a reference, and the input files were the original FASTA format nucleotide sequence files and gff3 format annotation files. Nucleotide diversity (PI) was calculated using DnaSP v6 software [38], with the window length set to 600 bp and the step size set to 200 bp.

### Ka/Ks analysis

We calculated Ka, Ks, and Ka/Ks ratios of homologous protein-coding genes in the chloroplast genomes of *A. selengensis* and eight other *Asteraceae* species, including intra-genus species (*A. argyi, A. annua, A. absinthium, A. borotalensis*) and intergeneric species (other genera of *Asteraceae*: *A. carlinoides, Chrysanthemum vestitum, Aster albescens, Helianthus carnosus*). The GenBank file was downloaded from NCBI, the protein-coding sequences in the GenBank file were extracted, and the homologous protein sequences were obtained by comparing other protein sequences with the reference protein sequences using BlastN (v2.10.1) to find the best match; then the homologous protein sequences were automatically aligned using MAFFT (v7.427) software [39], and the aligned protein sequence was mapped to the coding sequence to obtain the aligned coding sequence. Finally, the KaKs_Calculator2 software [40] was used to calculate the non-synonymous substitution rate (Ka), the synonymous substitution rate (Ks), and their ratios using the YN method. Ka/Ks > 1 indicates positive selection, Ka/Ks < 1 indicates purifying selection, and Ka/Ks = 1 denotes neutral selection.

### Phylogenetic analysis

The complete chloroplast genome GenBank data of 27 published genera of *Artemisia* and other genera in the Asteraceae family were downloaded from NCBI and phylogenetically analyzed with the 10 *A. selengensis* materials in this study, and the species names and GenBank accession numbers of the chloroplast genomes downloaded from NCBI are listed in Table S2 (**Supplementary Table S2**). Protein-coding sequences homologous and non-coding regions to the chloroplast genome were extracted for phylogenetic tree construction. The shared protein-coding sequences and non-coding regions were extracted using PhyloSuite software [41], and sequence alignment were performed using MAFFT. The compared sequences were then trimmed and concatenated and finally imported into IQTree to find the best model and construct a phylogenetic tree using the maximum likelihood method.

## Results

### Chloroplast genome structure and features

**Fig. 1:**
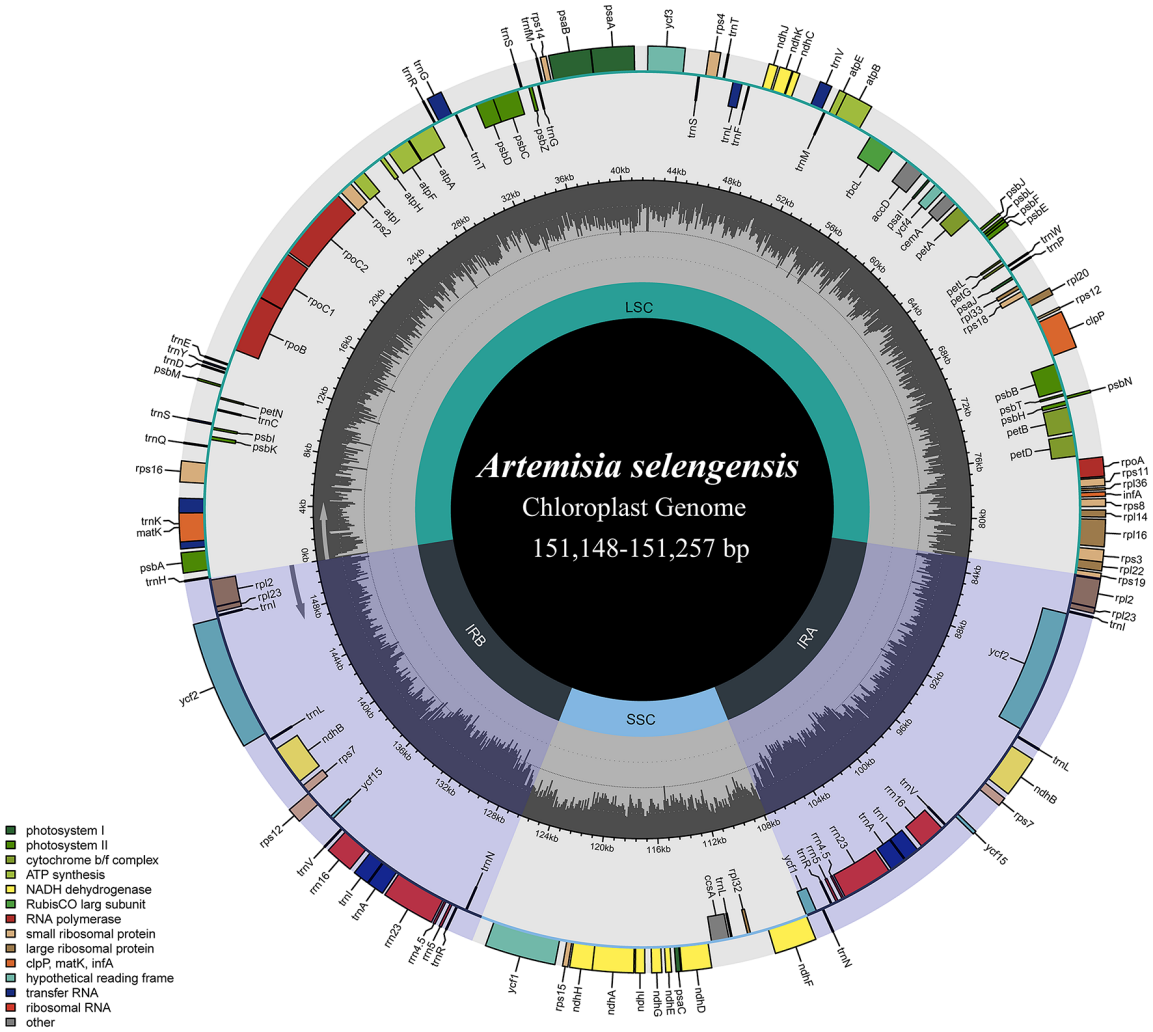
Complete chloroplast genome map of *A. selengensis*. In the lower-left corner is the legend, which classifies chloroplast genes according to their different functions. Genes on the outside of the circles are transcribed in a counterclockwise direction, and those on the inside of the circles are transcribed in a clockwise direction. The inner circle’s dark gray represents the distribution of GC content, while the outside circle’s light gray represents the distribution of AT content

In terms of gene content, gene order, and the number of gene introns, the 10 *A. selengensis* materials included in this study’s research had substantially conserved chloroplast genomes. The genome lengths ranged from 151,148 to 151,257 bp, and all had a typical tetrameric loop structure containing four regions, LSC, SSC, IRA, and IRB (Fig. 1). the LSC region was 82,888 to 82,956 bp in length, the SSC region was 18,338 to 18,390 bp in length, and the IR region was 24,961 to 24,964 bp in length. The total chloroplast genome GC content of the 10 materials was about 37.5%, showing a high degree of similarity. However, the GC content differed among the three major regions of the chloroplast genome, and the GC content of the IR region was 43.1%, which was higher than that of the LSC region (35.6%) and the SSC region (30.8%) (Supplementary Table S3).

Each chloroplast genome contains 133 genes, the number of protein-coding genes, tRNA genes, and rRNA genes are 88, 37, and 8, respectively (Table 2, Table SS3). The chloroplast genome contained 17 intron genes, including 11 protein-coding genes and 6 tRNA genes. Fifteen genes contained one intron and 2 genes (*ycf3* and *clpP*) contained two introns (Table 3). the LSC location included 68 protein-coding genes and 28 tRNA genes, the IR region contained 8 protein-coding genes, 8 tRNA genes, and 8 rRNA genes, and the SSC region contained 12 protein-coding genes and 1 tRNA gene. The genes in the LSC region accounted for 72.2% of the chloroplast genome, the IR region for 18.0%, and the SSC region for 9.8%.

**Table 2:** Predicted genes in the chloroplast genome of *A. selengensis*

Category	Gene group	Gene name
Photosynthesis	Subunits of photosystem I	*psaA*, *psaB*, *psaC*, *psaI*, *psaJ*
	Subunits of photosystem II	*psbA*, *psbB*, *psbC*, *psbD*, *psbE*, *psbF*, *psbH*, *psbI*, *psbJ*, *psbK*, *psbL*, *psbM*, *psbN*, *psbT*, *psbZ*
	Subunits of NADH dehydrogenase	*ndhA*^a^, *ndhB*^ad^, *ndhC*, *ndhD*, *ndhE*, *ndhF*, *ndhG*, *ndhH*, *ndhI*, *ndhJ*, *ndhK*
	Subunits of cytochrome b/f complex	*petA, petB*^*a*^, *petD*^*a*^, *petG, petL, petN*
	Subunits of ATP synthase	*atpA, atpB, atpE, atpF*^*a*^, *atpH, atpI*
	Large subunit of rubisco	*rbcL*
Self-replication	Proteins of large ribosomal subunit	*rpl14, rpl16*^*a*^, *rpl2*^*ad*^, *rpl20, rpl22, rpl23*^d^, *rpl32, rpl33, rpl36*
	Proteins of small ribosomal subunit	*rps11, rps12*^bd^, *rps14, rps15, rps16*^a^, *rps18, rps19, rps2, rps3, rps4, rps7*^d^, *rps8*
	Subunits of RNA polymerase	*rpoA, rpoB, rpoC1*^*a*^, *rpoC2*
	Ribosomal RNAs	*rrn16S*^d^, *rrn23S*^d^, *rrn4.5S*^d^, *rrn5S*^d^
	Transfer RNAs	*trnA-UGC*^*ad*^, *trnC-GCA, trnD-GUC, trnE-UUC, trnF-GAA, trnG-GCC, trnG-UCC*^*a*^, *trnH-GUG, trnI-CAU*^d^, *trnI-GAU*^*ad*^, *trnK-UUU*^*a*^, *trnL-CAA*^d^, *trnL-UAA*^*a*^, *trnL-UAG, trnM-CAU, trnN-GUU*^d^, *trnP-UGG, trnQ-UUG, trnR-ACG*^d^, *trnR-UCU, trnS-GCU, trnS-GGA, trnS-UGA, trnT-GGU, trnT-UGU, trnV-GAC*^d^, *trnV-UAC*^*a*^, *trnW-CCA, trnY-GUA, trnfM-CAU*
Other genes	Maturase	*matK*
	Protease	*clpP* ^b^
	Envelope membrane protein	*cemA*
	Acetyl-CoA carboxylase	*accD*
	c-type cytochrome synthesis gene	*ccsA*
	Translation initiation factor	*infA*
Genes of unknown function	Conserved hypothetical chloroplast ORF	*ycf1*^d^, *ycf15*^d^, *ycf2*^d^, *ycf3*^b^, *ycf4*

*Notes* Gene^a^:Gene with one introns Gene^b^:Gene with two introns; Gene^c^:Pseudo gene Gene^d^:Number of copies of multi-copy genes

**Table 3:** Location and length of exon and intron genes in the chloroplast genome of *A. selengensis*

Gene name	Gene location			Length (bp)				
	Strand	Start	End	Exon I	Intro I	Exon II	Intro II	Exon III
*rps16*	-	5213	6313	40	876	185		
*rpoC1*	+	15,982	18,783	434	729	1639		
*atpF*	+	26,694	27,947	145	699	410		
*ycf3*	-	41,857	43,805	124	702	230	740	153
*clpP*	-	68,744	70,742	71	800	291	611	226
*petB*	+	73,664	75,057	6	746	642		
*petD*	+	75,246	76,403	8	675	475		
*rpl16*	-	79,866	81,292	9	1019	399		
*rpl2*	-	83,005	84,494	397	662	431		
*ndhB*	-	93,043	95,245	777	670	756		
*ndhA*	-	117,585	119,752	553	1076	539		
*ndhB-2*	+	138,792	140,994	777	670	756		
*rpl2-2*	+	149,543	151,032	397	662	431		
*trnK-UUU*	-	1726	4365	37	2568	35		
*trnG-UCC*	-	29,957	30,755	23	729	47		
*trnL-UAA*	+	46,602	47,106	37	418	50		
*trnV-UAC*	-	51,055	51,702	38	573	37		
*trnI-GAU*	+	100,769	101,621	43	775	35		
*trnA-UGC*	+	101,686	102,570	38	812	35		
*trnA-UGC-2*	-	131,467	132,351	38	812	35		
*trnI-GAU-2*	-	132,416	133,268	43	775	35		

### IR boundary analysis

**Fig. 2:**
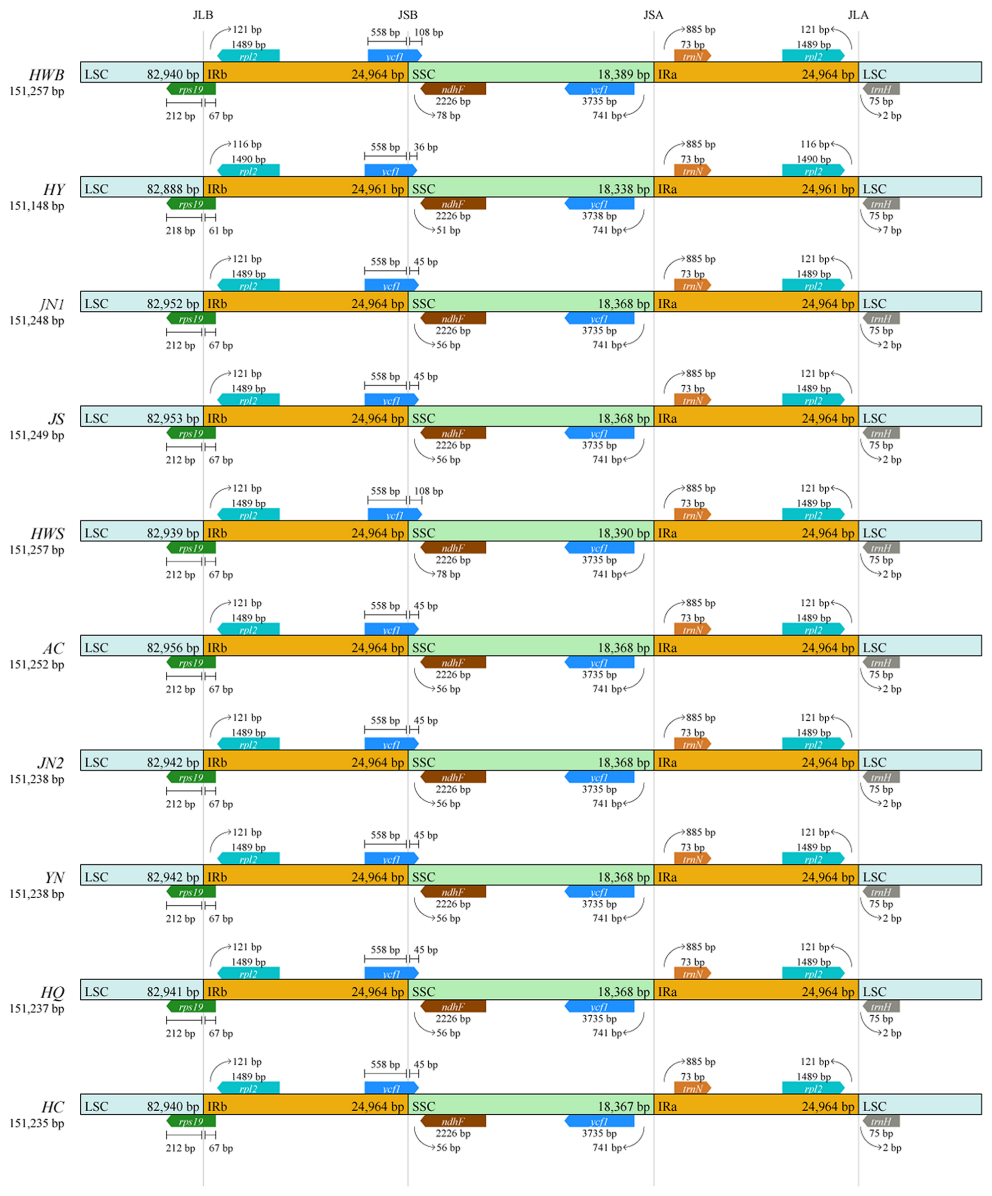
Comparison of chloroplast genome boundary regions of 10 *A. selengensis*

The IR region is one of the most conserved regions in the plant chloroplast genome, and the contraction and expansion of the IR region is the main cause of changes in chloroplast genome size and gene number, as well as a common evolutionary event in the chloroplast genome [35, 36]. Therefore, we performed boundary region analysis of the chloroplast genomes of 10 *A. selengensis* materials, and the results showed that the boundaries of the four regions were relatively conserved, and the types and numbers of genes in the boundary regions were highly consistent (Fig. 2). The contraction and expansion of the reverse repeat region showed high similarity at the boundary junctions of LSC/IRB, IRB/SSC, SSC/IRA, and IRA/LSC. The boundary of LSC/IRB was located at the *rps19* gene, which was 212–218 bp in the LSC region and 61–67 bp in the IRB region. The *ycf1* and *ndhF* genes were located at the SSC/IR region boundary, and the IRB/SSC boundary is located in the *ycf1* gene, which extends 558 bp into the IRB region and 36–108 bp into the SSC region. The *trnN-GUU* genes are all located in the IRA region at the SSC/IRA boundary, *rpl2* genes are completely present in the IRB region at the LSC/IRB boundary, and the *trnH-GUG* gene was located in the LSC region. In addition, we also found that the *ycf1* and *ndhF* genes overlapped at the IRB/SSC boundary in the *A. selengensis* material (HWB) from Baishazhou (HWB) and Shamao (HWS) in Wuhan City, Hubei Province. The location and order of the genes in the border area were largely constant in all materials, showing that the IR region is highly conserved.

### Codon usage analysis

**Fig. 3:**
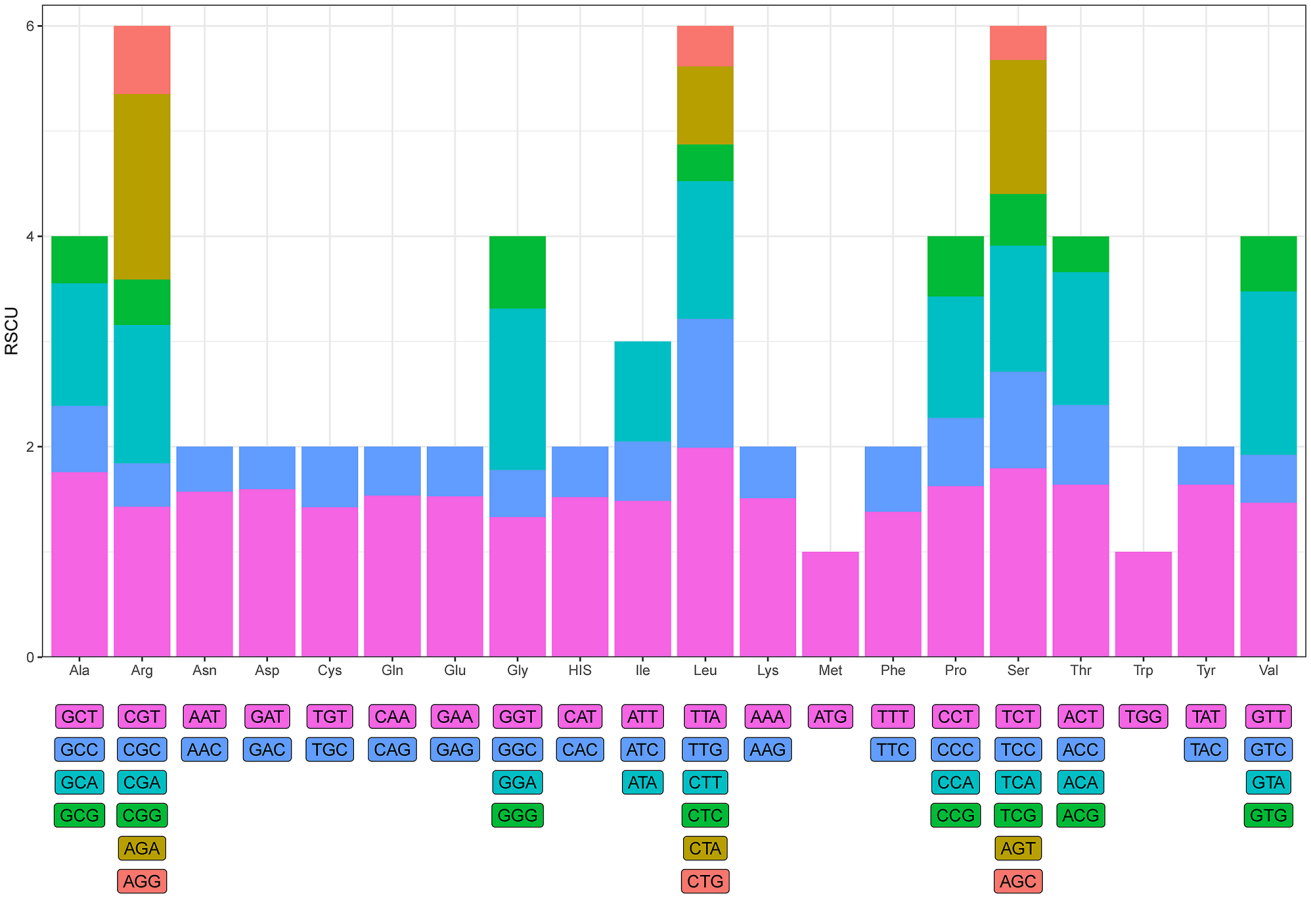
The amino acids’ Codon content encodes proteins in the chloroplast genome of *A. selengensis*

The base composition and GC content of the chloroplast genomes of the 10 *A. selengensis* materials in this study were identical, and we selected *A. selengensis* materials from Baishazhou, Wuhan, Hubei Province, to analyze the frequency of codon usage. The analysis of codon usage showed that a total of 20 amino acids (excluding stop codons) were encoded, with a frequency of 1.1-10.8% for each amino acid. Arginine, leucine, and serine were the most abundant amino acids, and cysteine was the least used (1.1%). Among the amino acids encoded, all amino acids were encoded by two to six codons except methionine and tryptophan, which were encoded by one codon (Fig. 3). The results showed that arginine, leucine, and serine were all encoded by six synonymous codons, of which the most frequently used codons were CGT, TTA, and TCT. Four synonymous codons were used to encode the amino acids alanine, glycine, proline, threonine, and valine. The most common codons were GCT, GGT, CCT, ACT, and GTT. Isoleucine contained three synonymous codons, with ATT being the most commonly used codon. Asparagine, aspartic acid, cysteine, glutamine, glutamate, histidine, lysine, phenylalanine, and tyrosine were encoded by two synonymous codons that were employed at about the same frequency. The most frequently used codons for each of these nine amino acids were AAT, GAT, TGT, CAA, GAA, CAT, AAA, TTT, and TAT. In the chloroplast genome of *A. selengensis*, there were 29 codons with RSCU > 1, 28 of which ended in A/T, accounting for 96.55%. 30 codons with RSCU < 1, 28 of which ended in G/C, accounting for 90.63%. The starting codons AUG and UGG were both unbiased codons (RSCU = 1). The findings revealed that the common base in the third position of the codon in *A. selengensis*’ chloroplast genome was A/T. The ATG codon used methionine encoding formylmethionine as the start codon. This is the most common start codon in the chloroplast genomes of all species. However, other codons have been found as start codons during translation, such as GTG (*rps19*), and ACG (*psbL*).

To clarify the codon usage bias of the chloroplast genomes in ten *A. selengensis* samples, ENC-plot analysis, PR2-plot analysis, and neutrality plot analysis were conducted concurrently. As indicated in Table 4, the overall GC content (GCall) of the chloroplast protein-coding genes in the ten *A. selengensis* samples is essentially the same, with the GC content at the first (GC1), second (GC2), and third (GC3) codon positions all being less than 50%. There are differences in the GC content across these three positions, with a decreasing trend observed from the first to the third position, indicating a pattern of GC1 > GC2 > GC3. This indirectly reflects a predominance of A and T bases in the chloroplast genomes of the ten *A. selengensis* samples, with the bases at the third codon position generally being A or T. As observed in Table 4, the average ENC (Effective Number of Codons) values for ten *A. selengensis* samples ranged from 49.018 to 49.035, all significantly higher than 35%, indicating that the overall codon bias in the chloroplast genomes is relatively weak.

**Table 4:** Analysis of codon usage of 10 chloroplast genome protein coding sequences of *A. selengensis*

Species	Genbank ID	GCall	GC1	GC2	GC3	GC3s	ENC	CdsCount
HWB	ON931227	37.74%	46.25%	38.10%	28.87%	24.67%	49.028	53
HWS	ON921081	37.76%	46.25%	38.13%	28.90%	24.75%	49.018	53
HQ	ON931228	37.74%	46.25%	38.10%	28.87%	24.67%	49.031	53
HY	ON942234	37.74%	46.25%	38.10%	28.87%	24.67%	49.032	53
HC	ON942235	37.74%	46.25%	38.10%	28.87%	24.68%	49.033	53
JN1	ON960153	37.74%	46.25%	38.10%	28.86%	24.66%	49.025	53
JN2	ON960154	37.74%	46.25%	38.10%	28.87%	24.67%	49.035	53
AC	ON968863	37.74%	46.25%	38.10%	28.87%	24.67%	49.035	53
JS	ON968864	37.74%	46.25%	38.10%	28.87%	24.67%	49.028	53
YN	ON968865	37.74%	46.25%	38.10%	28.87%	24.67%	49.028	53

### ENC-plot analysis

**Fig. 4:**
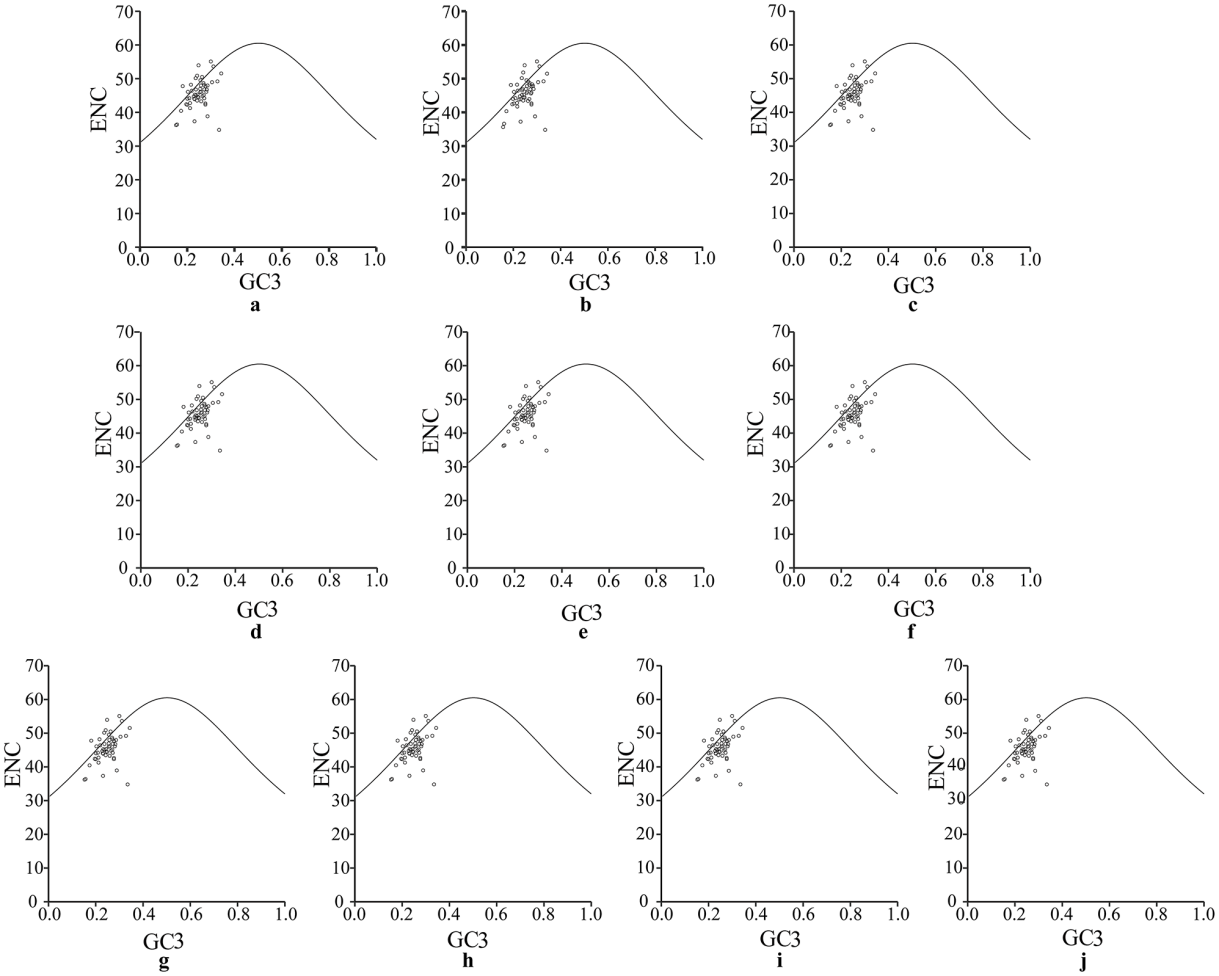
ENC-plot analysis of 10 *A. selengensis* chloroplast genomes. *Note* a ~ j *A. selengensis* germplasms in the figure are Wuhan Baishazhou, Wuhan Shamao, Qichun, Hunan Yueyang, Hunan Chenzhou, Jiangsu Nanjing 1, Jiangsu Nanjing 2, Anhui Chuzhou, Jiangxi Shangrao and Yunnan

Figure 4 reveals a high degree of consistency in the ENC-plot results for the chloroplast genomes of ten *A. selengensis* samples. Most genes are distributed on both sides of the curve, with a greater concentration of genes below the curve. The distribution of some genes along the standard curve indicates that their actual ENC values are close to the expected ones, suggesting that base mutations play a primary role in influencing codon usage bias. Conversely, genes deviating from the standard curve show significant differences between their actual and expected ENC values, indicating that in addition to the influence of base mutations, natural selection also affects codon usage bias.

### PR2-plot analysis

**Fig. 5:**
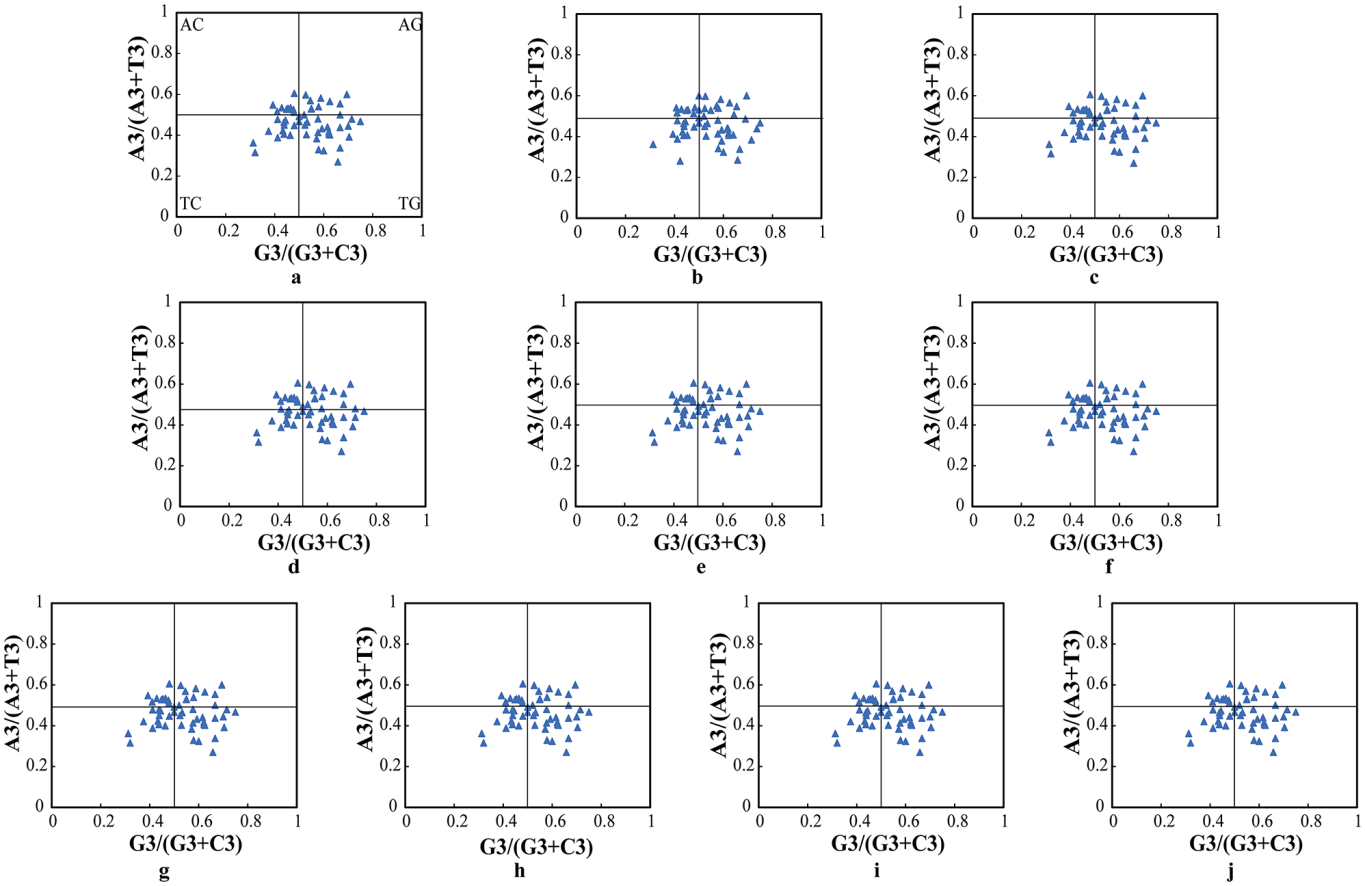
PR2-plot analysis of 10 *A. selengensis* chloroplast genomes. **a**–**j** *A. selengensis* germplasms in the figure are Wuhan Baishazhou, Wuhan Shamao, Qichun, Hunan Yueyang, Hunan Chenzhou, Jiangsu Nanjing 1, Jiangsu Nanjing 2, Anhui Chuzhou, Jiangxi Shangrao and Yunnan

From Fig. 5, it is evident that genes are randomly distributed across four regions, with most points situated far from the center, and the majority located in the bottom-right TG region. This overall distribution indicates a scenario where the third position base of codons is predominantly T > A and G > C, suggesting that the bias in the usage of the third base of codons in the chloroplast genomes of the ten *A. selengensis* samples is mainly influenced by natural selection.

### Neutrality plot analysis

**Fig. 6:**
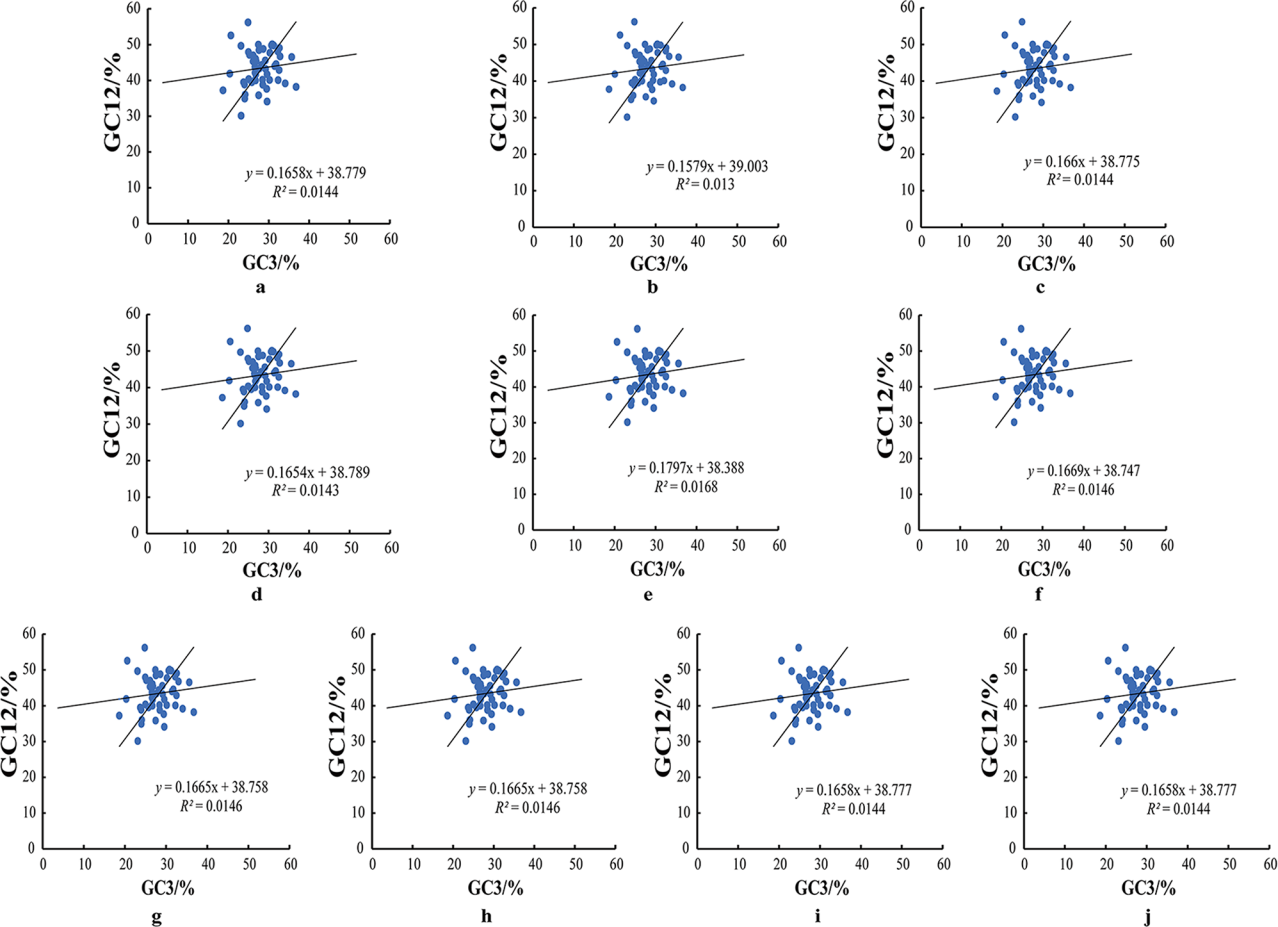
Neutrality plot analysis of 10 *A. selengensis* chloroplast genomes. **a**–**j** *A. selengensis* germplasms in the figure are Wuhan Baishazhou, Wuhan Shamao, Qichun, Hunan Yueyang, Hunan Chenzhou, Jiangsu Nanjing 1, Jiangsu Nanjing 2, Anhui Chuzhou, Jiangxi Shangrao and Yunnan

To analyze the impact on codon usage bias in the chloroplast genomes of ten *A. selengensis* samples, the average value of GC content at the first and second positions of codons (GC12) was plotted on the y-axis, and the GC content at the third position (GC3) was plotted on the x-axis. As indicated in Fig. 6, the GC12 values of the codons in the chloroplast genomes of the ten *A. selengensis* samples mainly range between 30.14 and 56.2, while the GC3 values are distributed between 23.03 and 25.55, demonstrating a higher frequency of A/T base usage at the third codon position. The slope of the fitted curve ranges from 0.1579 to 0.1797, with a regression coefficient (R^2^) greater than 0, indicating a positive correlation between GC12 and GC3, albeit not significant. This also suggests that the codon usage in the chloroplast genomes of the ten *A. selengensis* samples differs between the mutations patterns at the first and second positions and the third, with the codon usage bias at the first and second positions being more influenced by natural selection.

### Scattered repeat sequence and microsatellite (SSR) analysis

**Fig. 7:**
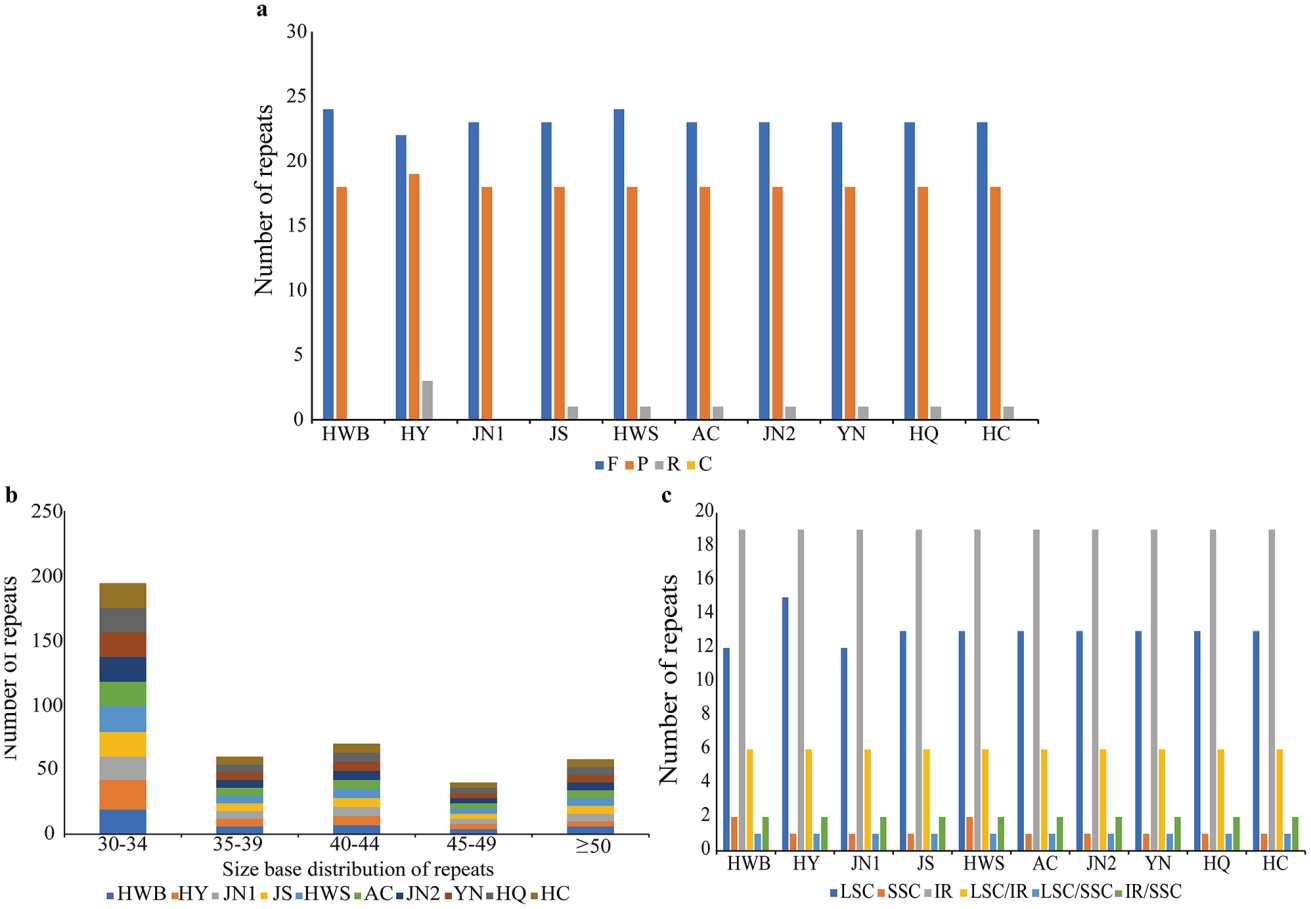
Type and distribution of scattered repeats in 10 *A. selengensis*. (**a**) Types and numbers of the four repeats. (**b**) Distribution of the sizes of the repetitive sequences. (**c**) Distribution of repetitive sequences in the three major regions of the chloroplast genome

Scattered repetitive sequences can be generally classified into four types: forward repeats, reverse repeats, complementary repeats, and palindromic repetitive sequences. In this study, 42–45 repetitive sequences were detected in 10 materials by REPuter, which contained 22–24 forward repeats, 19–20 palindromic repeats, and 0–3 reverse repeats, and no complementary repeats were detected in all materials. The abundance of repetitive sequences varied depending on the repetitive sequence type, with forward repeats exhibiting the greatest abundance among all materials, followed by palindromic repeats (Fig. 7a). The minimum length of these repetitive sequences was at least 30 bp and the maximum was 72 bp, and most of the repetitive sequences were distributed in the range of 30–44 bp in length (Fig. 7b). Among the four regions, the IR region contained more repetitive sequences than the LSC and SSC, while some repetitive sequences were also shared between different regions of the chloroplast genome (Fig. 7c).

**Fig. 8:**
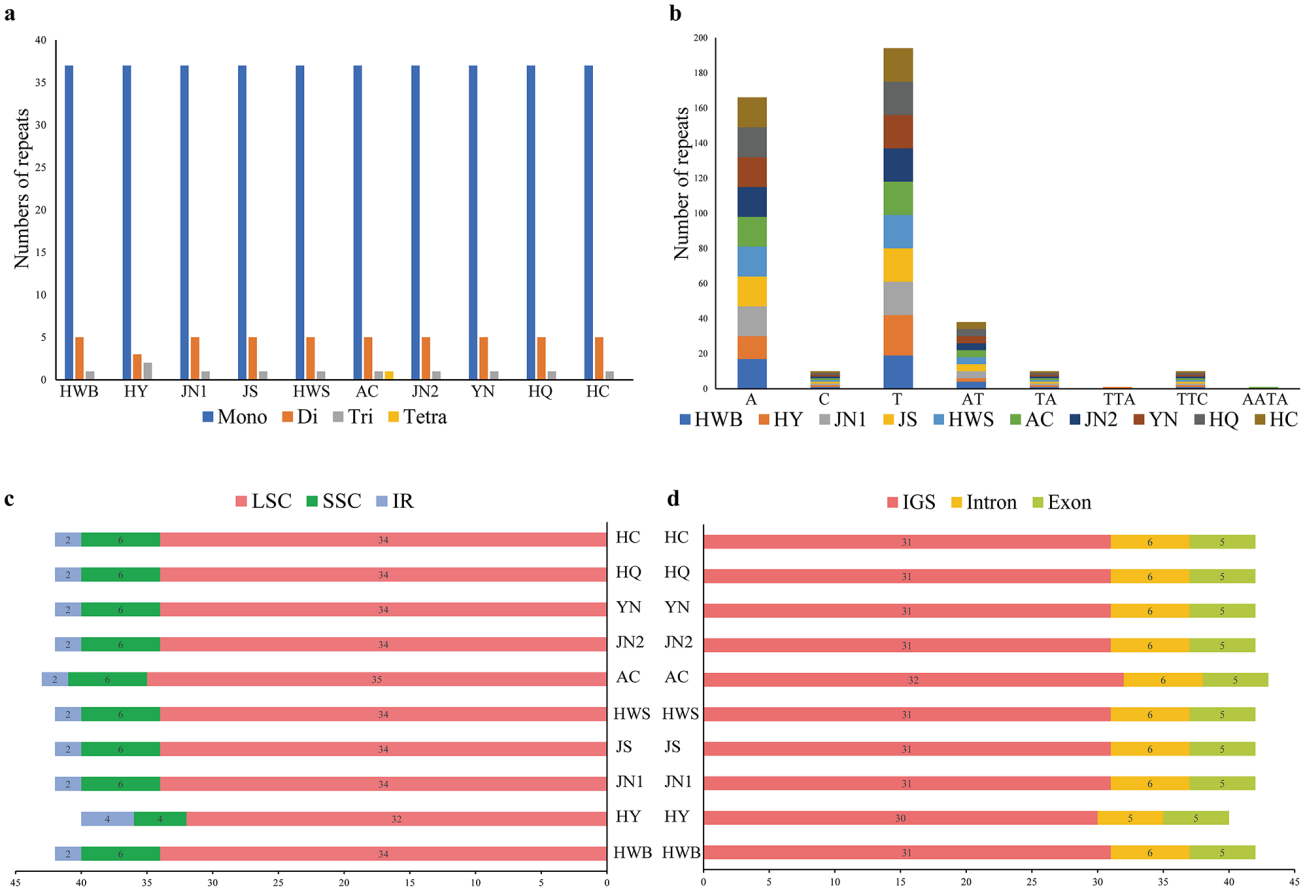
Type and distribution of chloroplast genomic SSRs in 10 *A. selengensis*. (**a**) Number of SSR repeat types. (**b**) Number of identified SSR motifs in different repeat types. (**c**) Frequency of SSR occurrence in LSC, SSC, and IR regions. (**d**) Percentage distribution of SSRs in IGS, Intron, and Exon

The chloroplast genomes of 10 materials were detected with SSR loci using MISA. The results showed that the number of cpSSRs ranged from 42 to 44 (Fig. 8a), with the least number of SSR loci detected for *A. selengensis* (HY) from Yueyang City, Hunan Province, with 42 SSR loci, the most SSR loci detected for *A. selengensis* (AC) from Chuzhou City, Anhui Province, with 44 loci, and 43 SSR loci detected for the remaining eight materials. Single nucleotide repeats were the most abundant in all materials, consisting mainly of polyadenine (*ploy-A*) and polythymine (*ploy-T*), followed by dinucleotide repeats AT/TA, and rarely containing tandem guanine (G) or cytosine (C) (Fig. 8b). In addition, we did not find a large number of trinucleotide to tetranucleotide repeats. SSR repeats were randomly distributed in the chloroplast genome (Fig. 8c), with 339 in LSC, 58 in SSC, and 22 in IR regions (310 in spacer regions, 59 in introns, and 50 in exons). The results showed that most of the SSRs were distributed in the LSC region and the spacer region (Fig. 8d).

### Comparative genomic analysis

**Fig. 9:**
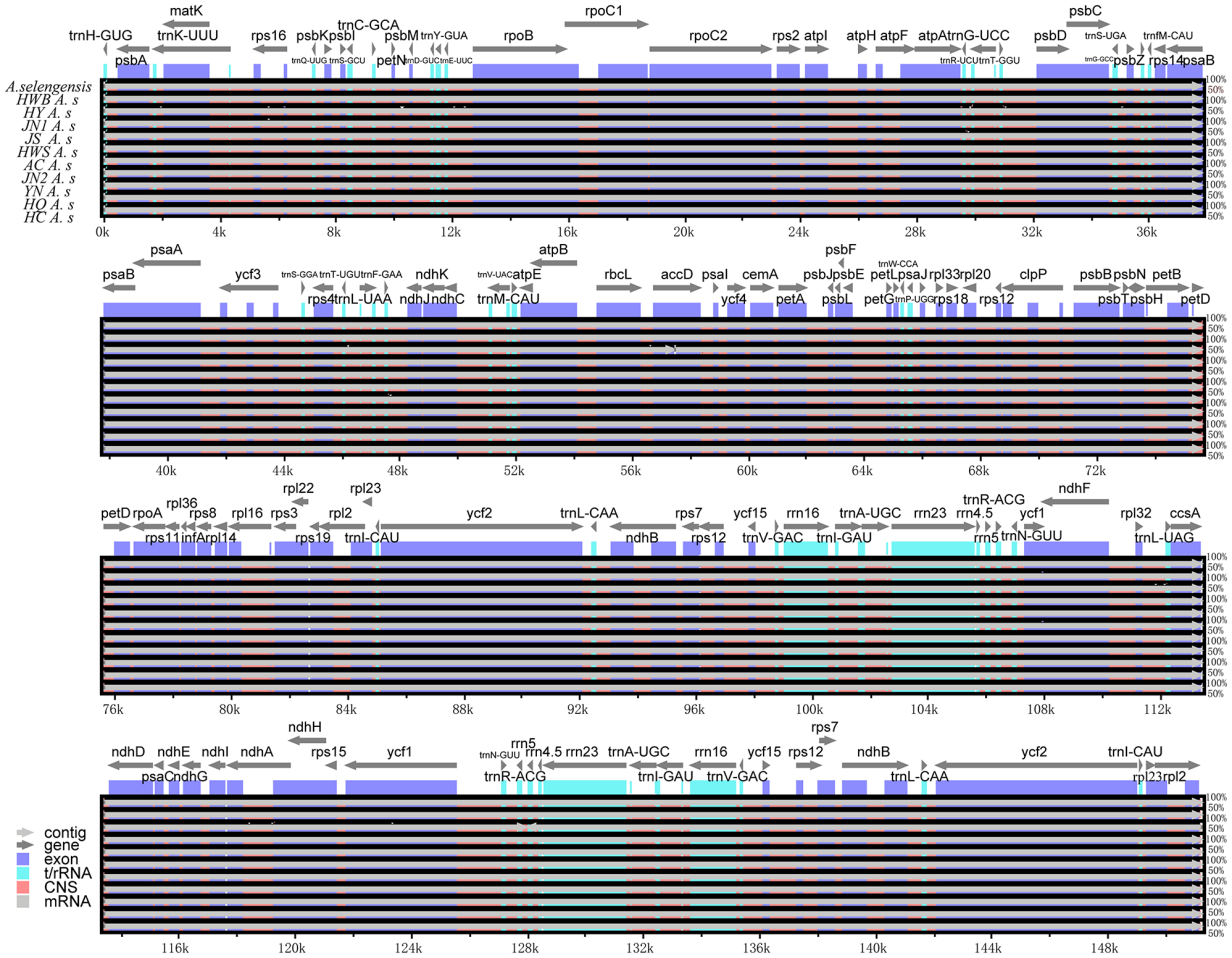
Sequence alignment analysis of the chloroplast genomes of 10 *A. selengensis* in this study using mVISTA software, using the Dongting Lake chloroplast genome sequence as a reference. The x-axis represents the coordinates of the chloroplast genome. The y-axis indicates the average percentage of identity within 50–100%. The direction of gene transcription is indicated by gray arrows, and the genomic regions are color coded for exons, tRNA, rRNA, conserved non-coding sequences, and mRNA

In this study, we used the online software mVISTA to perform sequence alignment analysis of the complete chloroplast genomes of ten *A. selengensis* materials, using the NCBI-published Dongting Lake *A. selengensis* (NC_039647) as a reference. The results showed that the nucleotide sequence similarity of the ten chloroplast genomes was extremely high, and the genomes showed a high degree of covariance with each other, indicating their evolutionary conservation at the genome level (Fig. 9). After comparative analysis, we found that IR regions and coding regions were more conserved than non-coding regions. More variation in the chloroplast genome occurs in the non-coding regions (CNS), where the non-coding regions with high levels of variation are *petN-psbM, trnR-UCU-trnG-UCC*, and *rpl32-trnL-UAG*, which can be used for phylogenetic studies [42, 43]. Variable regions have also been found in some coding genes, such as *accD, rps19*, and *ndhA*, and differences in *accD* and *ycf1* have been found in other angiosperm plastid genomes [24, 44–46], making these genes also reliable markers for phylogenetic analyses [47].

**Fig. 10:**
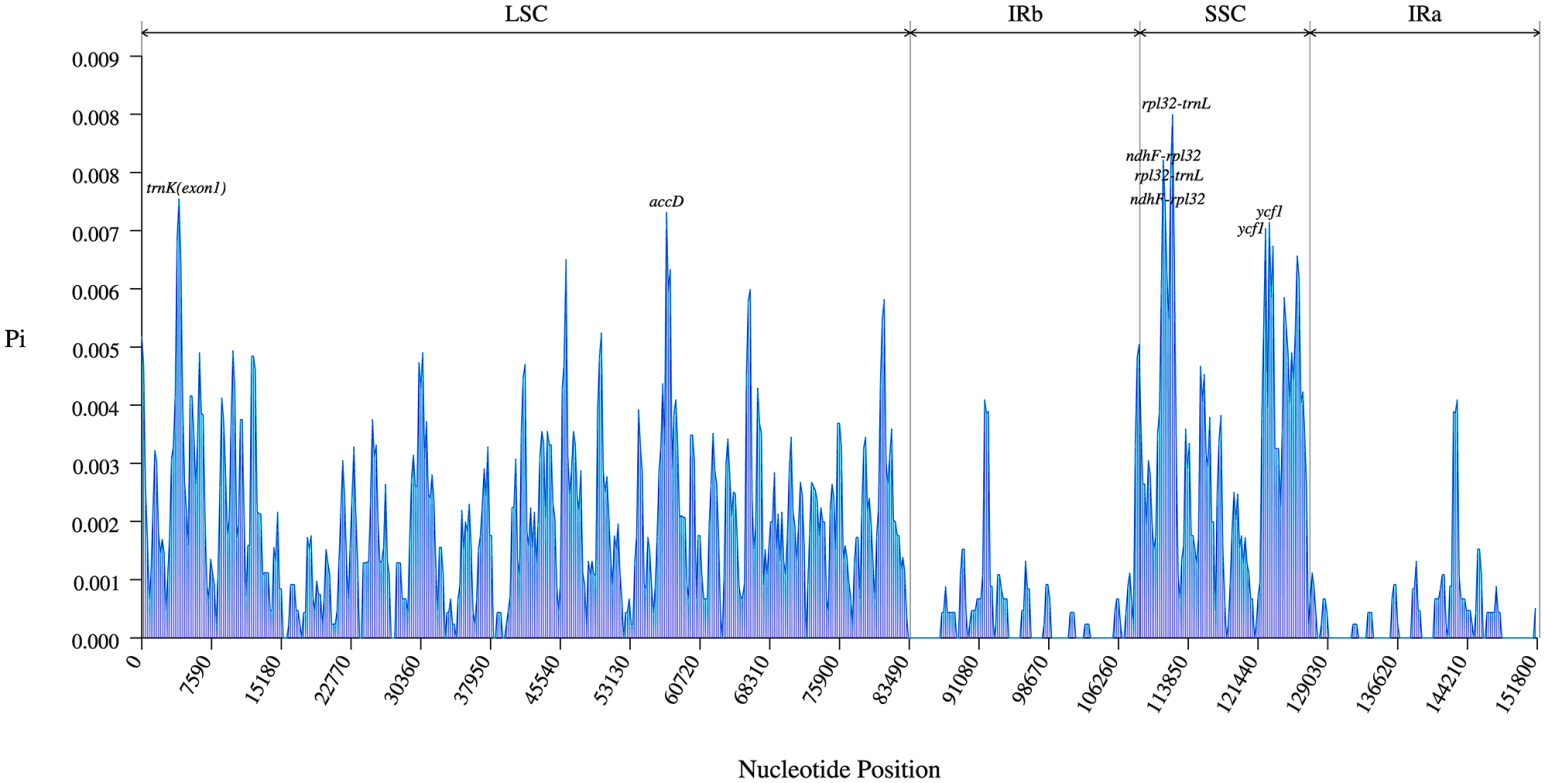
Sliding window analysis of the whole chloroplast genome. Sliding window analysis between 10 *A. selengensis* and other *Artemisia* species. Sliding window length: 600 bp; step size: 200 bp. X-axis: position of the sliding window. Y-axis: nucleotide diversity of each window

In addition, to further assess the sequence divergence of the chloroplast genome of *A. selengensis*, we calculated the nucleotide diversity of the chloroplast genome of 10 *A. selengensis* materials with other *Artemisia* species (*A. fukudo, A. annua, A. argyi, and A. capillaris*) using DNAsp software. The analysis indicated that the nucleotide diversity varied from 0 to 0.009 within a sliding window length of 600 base pairs, with the IR area having the lowest nucleotide diversity and the SSC region having the greatest nucleotide diversity. By analyzing the calculated nucleotide diversity, a total of eight mutation hotspot regions were detected (Fig. 10), while *rpl32-trnL, ndhF-rpl32*, and *ycf1* sequence intervals each had two mutation hotspot regions inside the interval. Among them, rpl32-trnL had the highest Pi value (Pi = 0.00844). This was followed by four other mutation hotspot regions, including *ndhF-rpl32, trnK(exon1), accD*, and *ycf1*; most of these mutation hotspots were located in the SSC region.

### Ka/Ks analysis of protein-coding genes

**Fig. 11:**
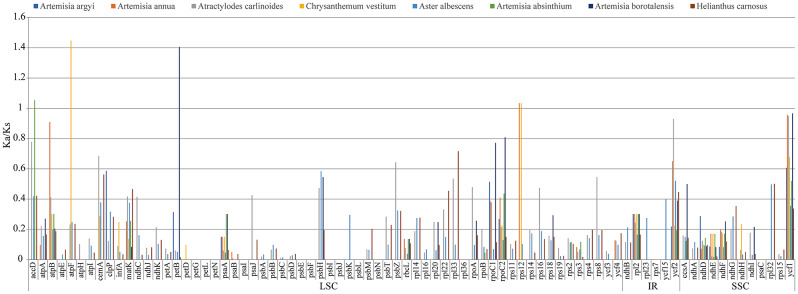
Ka/Ks analysis of homologous protein-coding genes in the complete chloroplast genomes of eight *Asteraceae* species and *A. selengensis*

Low rates of evolution were seen in the rates of synonymous substitutions (Ks), non-synonymous substitutions (Ka), and their ratios (Ka/Ks) in the chloroplast genome. Synonymous substitutions were more common than nonsynonymous substitutions (Fig. 11), thus, most genes had lower Ka/Ks values, which were affected by the effect of purifying selection. The average Ka value between *A. selengensis* and four *Artemisia* plants was 0.1359, and the average Ks value was 0.2313, according to the comparative study of homologous protein-coding genes; the average Ka value between *A. selengensis* and four other Asteraceae species genera was 0.6861 and the average Ks value was 4.1240. Meanwhile, we also calculated the Ka/Ks ratio to assess the effect of selective pressure on protein-coding genes. In this study we found that within the genus *Artemisia*, *accD, rps12*, and *petB* genes evolved under positive selection pressure with Ka/Ks > 1; between genera *atpF* gene and *rps12* gene with Ka/Ks > 1 showed positive selection pressure. No neutral selective effect was found (Supplementary Table S4).

#### Phylogenetic analysis

**Fig. 12:**
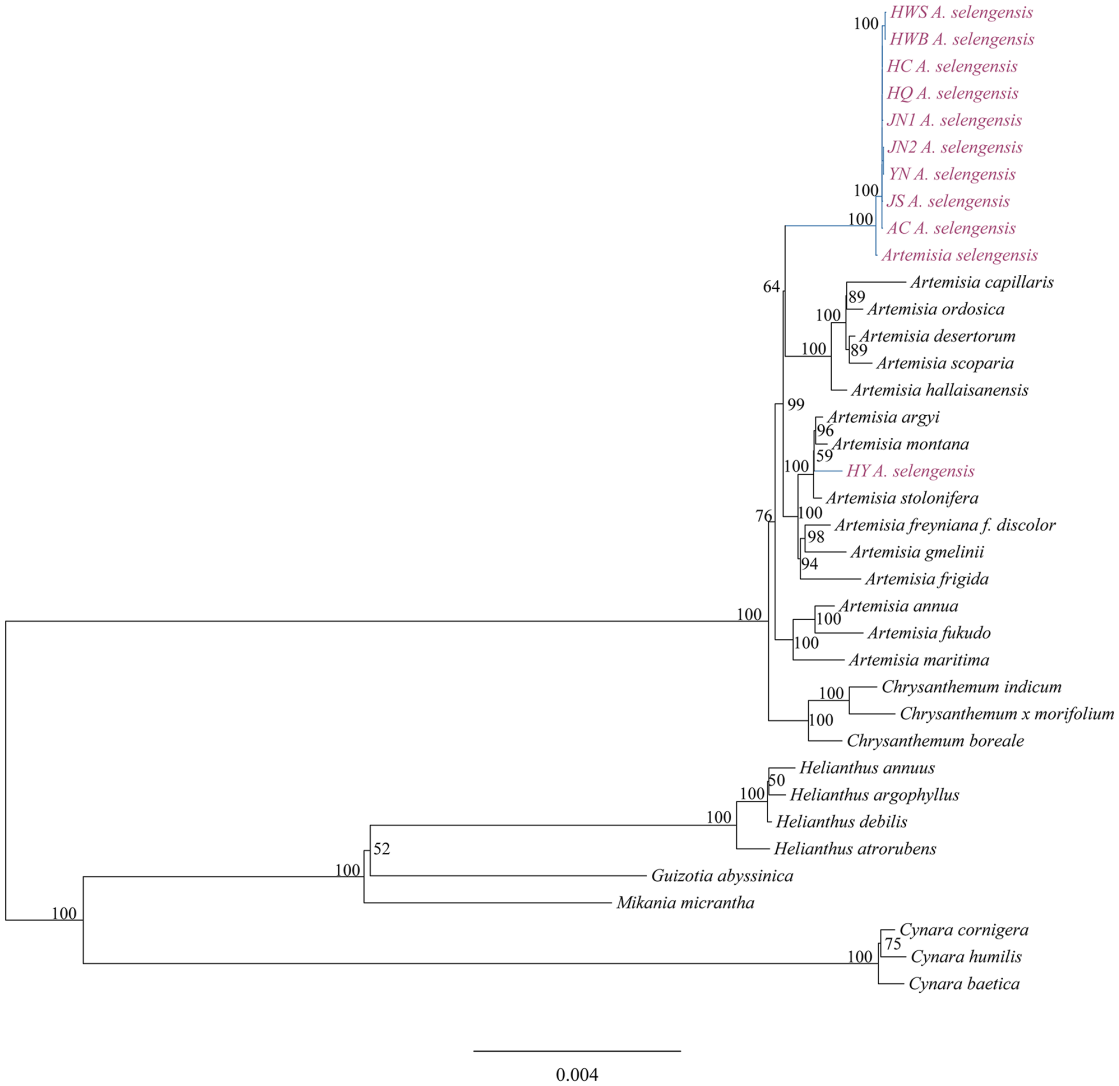
Phylogenetic tree construction using maximum likelihood method based on sequences of homologous protein-coding genes from 10 *A. selengensis* and 27 other *Asteraceae*

Using 81 homologous protein-coding sequences, we performed a phylogenetic analysis of 10 *A. selengensis* materials and 27 other Asteraceae plants by the maximum likelihood method with the best model of TVM + F + R2 (Fig. 12), and the results yielded well-supported tree topologies. Among the 10 materials we studied, except for *A. selengensis* (HY) from Yueyang, Hunan, which clustered into a small branch with *A. argyi, A. montana, and A. stolonifera*, the remainder of *A. selengensis* formed a minor branch on its own, then merged with other *Artemisia* species to create a monophyletic group that is sister to the genus *Chrysanthemum*.

**Fig. 13:**
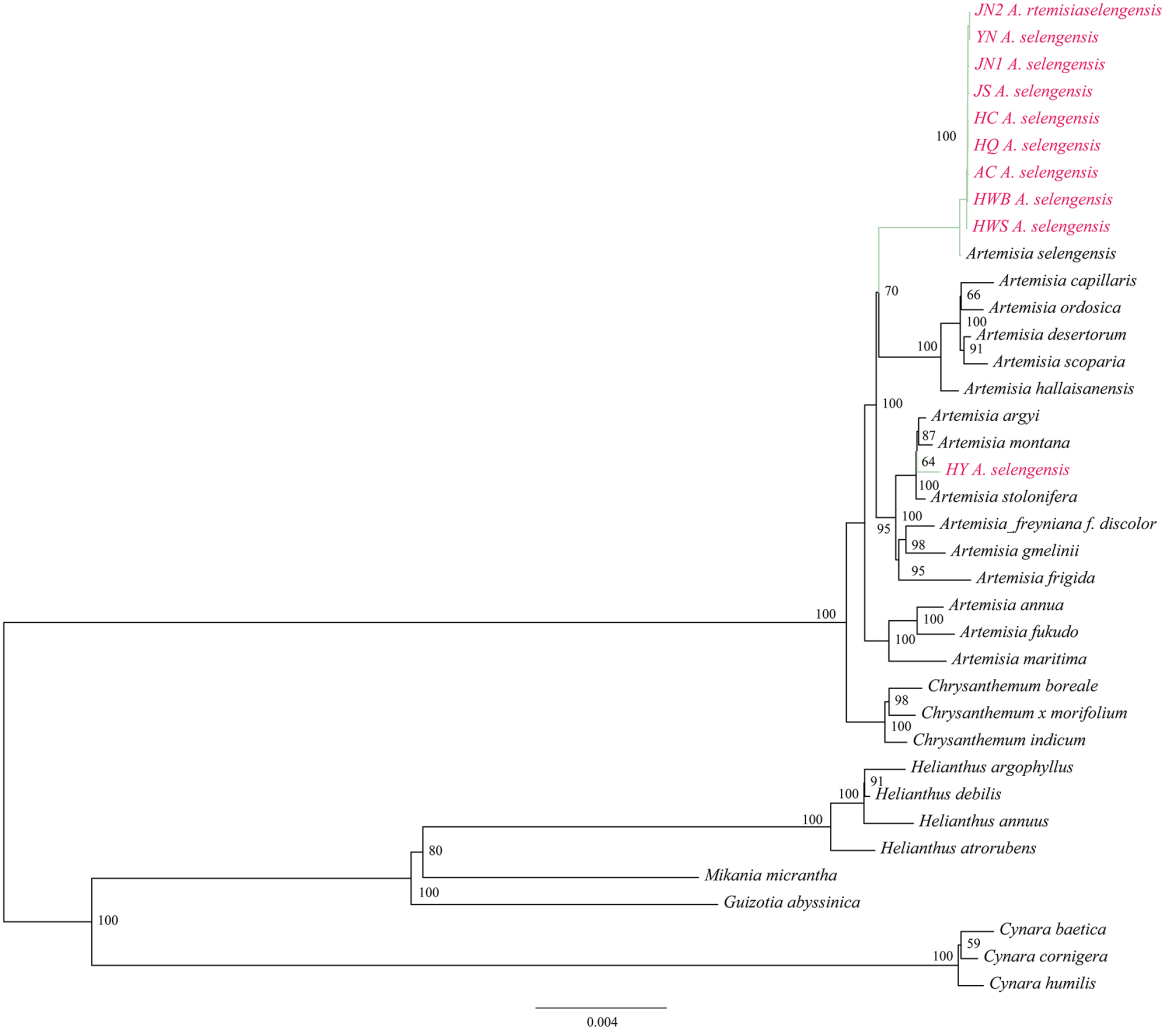
Phylogenetic tree construction using maximum likelihood method based on sequences of non-coding regions from 10 *A. selengensis* and 27 other *Asteraceae*

By extracting and comparing the non-coding regions of the complete chloroplast genomes, we conducted phylogenetic analyses using the maximum likelihood method on ten samples of *A. selengensis* and 27 other Asteraceae plants. The optimal model was identified as TVM + F + R2. The phylogenetic tree demonstrated well-supported branches (Fig. 13). Furthermore, it was observed that phylogenetic trees constructed from homologous protein-coding sequences and non-coding regions in the complete chloroplast genome sequences exhibited highly similar topologies and support rates.

## Discussion

In this study, we compared the chloroplast genome sequences of 10 *A. selengensis* materials from six provinces, which were very similar in terms of genome size, gene number, gene sequence, and GC content, and also similar to the chloroplast genome GC content of other *Artemisia* plants in the *Asteraceae* family, such as *A. annua* [26]. All chloroplast genomes were relatively uniform in length, ranging from 151,148 to 151,257 bp. All genomes encoded 133 genes, including 88 protein-coding genes (CDS), 37 tRNA genes, and 8 rRNA genes. GC content was highly conserved (37.5%), however, there were significant differences in GC content in different regions of the chloroplast genome, with the IR region having significantly higher GC content than the LSC and SSC regions, which may be due to four rRNA genes replicated in the IR region [48].

In addition, rRNA genes are more enriched in GC. The IR region of the chloroplast genome is more conserved than the LSC and SSC areas and plays a crucial function in maintaining the structure of the chloroplast genome and safeguarding critical genes [49, 50]. In our study, the chloroplast genome lengths of the 10 materials did not vary much, and the distribution of genes in the border regions was very similar with no significant changes. This indicates that the chloroplast genome of *A. selengensis* is highly conserved, consistent with the slow evolution of chloroplast genomes in other land plants [51].

Contraction and expansion of IR regions have an impact on the length and number of genes in the chloroplast genome of a species, leading to gene duplication, deletion, and pseudogene formation [52]. Comparative analysis of boundary regions can help in species’ phylogenetic and evolutionary analysis [53]. In this study, boundary analysis was performed on 10 *A. selengensis* chloroplast genomes, and it was found that the number and order of genes at the regional boundaries were highly conserved, and only the genes at the boundary junctions differed in their proximity to the boundary. Among them, *rps19* and *ycf1* were located at the boundaries of LSC/IRB and IRB/SSC respectively, while an overlapping region between *ycf1* and *ndhF* was found. In contrast, gene overlap between *ycf1* and *ndhF* was also found in *A. capillaris* [54] and *C. humilis* [55], which may be related to the contraction and expansion of the IR region.

Codons are key transmitters of genetic information and are used in the translation and synthesis of proteins from nucleic acids [56]. Also, codon use bias affects the quantity of gene expression, which is crucial for sustaining the life activities of the species, as well as the production of functional proteins [57] and also has an impact on the amount of gene expression, which is significant for maintaining the life activities of the species. In the present study, the codon usage bias of the 10 *A. selengensis* materials was essentially the same. The most used amino acids were arginine, serine, and leucine, with a preference for codons containing A and T bases, and with a preference for codons whose third bases ended in A and T. This result is consistent with the results of codon bias analysis of chloroplast genomes of other species [58–60]. However, other codons were found as start codons during translation, such as GTG (*rps19*) and ACG (*psbL*), which also occur in species of the family Araceae [61]. Through analysis of the GC content at the three codon positions, ENC-plot analysis, PR2-plot analysis, and neutrality plot analysis, it was observed that the overall GC content of the chloroplast genomes in ten *Artemisia* samples and the GC content at the three positions do not exceed 50%. Furthermore, the GC content decreases progressively from the first to the third position, with the overall A/T content being higher than G/C. In terms of codon usage, A/T is utilized more frequently, corresponding to the results of the RSCU (Relative Synonymous Codon Usage) analysis.

The type and number of repetitive sequences have a significant effect on chloroplast genome sequences, leading to differences in chloroplast genomes of different species [62]. We investigated the scattered repeats and microsatellite sequences in the chloroplast genomes of 10 *A. selengensis* materials, with 42–45 scattered repeats per material, most of which were positive repeats, and no complementary repeats were detected. Microsatellite repeats are randomly distributed in the chloroplast genome and are frequently used for species identification and phylogenetic analysis because they are co-dominant and highly polymorphic, making them effective markers for studying the genetics of plant populations [63, 64]. The number of SSRs we detected from each material ranged from 42 to 44, most commonly single nucleotide repeats, and most of the cpSSRs presented short polyadenines (*ploy-A*) or short polythymines (*ploy-T*) [65]. Most of the SSRs detected were distributed in the LSC region and the spacer region, in agreement with the results of the analysis of the genus *Atractylodes* [66]. The amount and distribution of repeated sequences in each material in this investigation were consistent, suggesting little variance and a low rate of variation across *A. selengensis* chloroplast genomes. These discovered repeated sequences may be employed as novel molecular markers in future genetic diversity investigations in *Artemisia* species.

In this study, chloroplast genome sequence alignment between 10 *A. selengensis* materials and Dongting Lake *A. selengensis* also showed high sequence identity and covariance, and some variable regions such as *petN-psbM*, *rpl32-trnL-UAG* were found in both non-coding and coding regions; by calculating nucleotide diversity, the nucleotide diversity values within a sliding window length of 600 bp ranged from 0 to 0.009, we identified eight mutational hotspot regions, including *rpl32-trnL, ndhF-rpl32, ycf1, ndhF-rpl32, trnK(exon1)* and *accD*. These mutational hotspots can be used as novel markers for subsequent phylogenetic analysis and DNA barcoding studies [67]. The role of selection pressure on protein-coding genes is also an important indicator for phylogenetic studies, in our study, *accD, rps12, petB*, and *atpF* genes evolved under the positive selection pressure of Ka/Ks > 1, but most genes had low Ka/Ks values and were affected by purifying selection, and no neutral selection was found in all genes.

The genus *Artemisia*, within the Asteraceae family, is comprised of roughly 400 species globally recognized for their adaptability, ecological significance, and medical value, notably *Artemisia annua*’s artemisinin for malaria treatment. Research on *Artemisia* contributes to our understanding of plant adaptability, evolution, and phylogeny, offering insights into species distribution and plant-environment interactions. Jiang [68] analyzed 71 protein-coding genes across 8 species of *Artemisia* and 13 species within the Asteraceae family, revealing that *A. stechmanniana* and *A. tangutica* share a close phylogenetic relationship, demonstrating a pronounced sisterhood bond. Concurrently, Jin et al. [69] utilized Bayesian inference (BI) and ML methods to construct a phylogenetic tree encompassing 38 *Artemisia* species. They found that the evolutionary branching of *Artemisia* was monophyletic, and all samples of the same species were clustered together; however, *Artemisia* belonging to various species and subspecies were not completely clustered together. Phylogenetic analysis based on a maximum likelihood method with 81 protein-coding sequences showed that all *A. selengensis* clustered together and then with other *Artemisia* species in the *Asteraceae* family to form the *Artemisia* monophyletic group, with *Artemisia* and *Chrysanthemum* as sister genera. This is consistent with the results of the chloroplast genome phylogenetic analysis of *A. maritima* and *A. absinthium* [70]. Phylogenetic trees constructed from alignments of non-coding region sequences showed consistent results with those based on protein-coding sequences in terms of topology and support rates. The genus *Artemisia* was confirmed as a monophyletic group, and identified as sister to the genus Chrysanthemum. This phenomenon may be associated with the inherent conservation of the chloroplast genome. Additionally, due to the relative stability of the chloroplast genome structure and the high collinearity of genes, both coding and non-coding regions exhibit synchronicity in their evolutionary patterns. In plant phylogenetic studies, the non-coding regions of the chloroplast genome can serve as an effective supplementary dataset. Analyzing these regions may enhance our understanding of the evolutionary dynamics of plant genomes and their adaptability to environmental changes, particularly when discerning differences among closely related species. Therefore, future research should delve into the phylogenetic potential of non-coding regions in various plant chloroplast genomes and explore how to effectively integrate data from both coding and non-coding regions to achieve a more comprehensive analysis of phylogenetic relationships.

## Conclusions

In summary, in this study, we assembled and annotated the complete chloroplast genomes of 10 *A. selengensis* germplasm resources using Illumina high-throughput sequencing data, and performed a comparative analysis of the chloroplast genome structure, which showed that the chloroplast genome structure of *A. selengensis* from different regions, with little difference in gene type and number, was relatively conserved. The overlap of *ycf1* and *ndhF* genes was identified by IR boundary analysis; the detected microsatellite (SSR) and differential hotspot areas discovered by whole genome sequence alignment analysis were beneficial for *Artemisia* population genetics and phylogenetic analysis. The phylogenetic analysis also clarified the evolutionary relationships among *Artemisia* species. This study has important implications for further studies on genetic diversity and evolutionary relationships in *Asteraceae*.

### Electronic supplementary material

Below is the link to the electronic supplementary material.

Supplementary Material 1

Supplementary Material 2

Supplementary Material 3

Supplementary Material 4

## Data Availability

The datasets generated during the current study are available in the NCBI repository, the persistent accession number of the dataset is as follows: ON931227, ON942235, ON968864, ON921081, ON968865, ON931228, ON960154, ON960153, ON942234, ON968863. At the same time, the BioProject ID of the whole genome sequencing data of *A. selengensis* chloroplast submitted to NCBI is PRJNA946329.

## Declarations

### Statement

Ten *A. selengensis* materials were taken from Wuhan, Hubei, Qichun, Yueyang, Chenzhou, Nanjing, Anhui, Chuzhou, Shangrao, Jiangxi, and Yunnan, respectively. The samples are stored in the Engineering Research Center for the Protection, Development, and Utilization of Characteristic Biological Resources in the Han River Basin of Hubei Province, School of Life Sciences, Jianghan University. These samples were identified by Professor Zeng Changli, School of Life Sciences, Jianghan University. I confirm that all *A. selengensis* materials have been collected with permission and permission, and the methods of material collection and experimental research have been conducted in accordance with relevant guidelines/regulations/legislation. The collection of *A. selengensis* materials complies with the International Union for the Conservation of Nature’s Policy Statement on Endangered Species Research and the Convention on Trade in Endangered Species of Wild Fauna and Flora.

## References

[1] Panero JL, Funk VA (2002). Toward a phylogenetic subfamilial classification for the Compositae (Asteraceae). Proc Biol Soc Wash.

[2] Fu Z-X, Jiao B-H, Nie B, Zhang G-J, Gao T-G (2016). A comprehensive generic-level phylogeny of the sunflower family: implications for the systematics of Chinese Asteraceae. J Syst Evol.

[3] Pandey AK, Singh P (2017). The Genus Artemisia: a 2012–2017 literature review on Chemical Composition, Antimicrobial, Insecticidal and antioxidant activities of essential oils. Med (Basel).

[4] Tu Y (2016). Artemisinin-A gift from traditional Chinese medicine to the World (Nobel lecture). Angew Chem-Int Edit.

[5] Hong-wei L (2018). Xi-du N. Analysis of Trace Elements in Wild Artemisia Selengensis using inductively coupled plasma Tandem Mass Spectrometry. Spectrosc Spectr Anal.

[6] Li R, Tao M, Xu T, Huang Y, Zogona D, Pan S (2022). Artemisia selengensis Turcz. Leaf extract promotes longevity and stress resistance in Caenorhabditis elegans. J Sci Food Agric.

[7] Shi F, Jia X, Zhao C, Chen Y (2010). Antioxidant activities of various extracts from Artemisisa Selengensis Turcz (LuHao). Molecules.

[8] Wang J, Han J, Lu Z, Lu F (2020). Preliminary structure, antioxidant and immunostimulatory activities of a polysaccharide fraction from Artemisia selengensis Turcz. Int J Biol Macromol.

[9] Wang S, Xie X, Zhang L, Hu Y, Wang H, Tu Z (2020). Inhibition mechanism of alpha-glucosidase inhibitors screened from Artemisia selengensis Turcz root. Ind Crop Prod.

[10] Jose Abad M, Miguel Bedoya L, Apaza L, Bermejo P (2012). The Artemisia L. Genus: a review of Bioactive essential oils. Molecules.

[11] Zhang L, Tu Z, Yuan T, Wang H, Fu Z, Wen Q (2014). Solvent optimization, antioxidant activity, and chemical characterization of extracts from Artemisia selengnesis Turcz. Ind Crop Prod.

[12] Du Z, Lu K, Zhang K, He Y, Wang H, Chai G (2021). The chloroplast genome of Amygdalus L. (Rosaceae) reveals the phylogenetic relationship and divergence time. BMC Genomics.

[13] Li C, Cai C, Tao Y, Sun Z, Jiang M, Chen L (2021). Variation and evolution of the whole chloroplast genomes of Fragaria spp. (Rosaceae). Front Plant Sci.

[14] Song W, Chen Z, He L, Feng Q, Zhang H, Du G (2022). Comparative Chloroplast Genome Analysis of Wax Gourd (Benincasa hispida) with three Benincaseae species, revealing Evolutionary dynamic patterns and phylogenetic implications. Genes.

[15] Feng S, Zheng K, Jiao K, Cai Y, Chen C, Mao Y (2020). Complete chloroplast genomes of four Physalis species (Solanaceae): lights into genome structure, comparative analysis, and phylogenetic relationships. BMC Plant Biol.

[16] Wang M, Wang X, Sun J, Wang Y, Ge Y, Dong W (2021). Phylogenomic and evolutionary dynamics of inverted repeats across Angelica Plastomes. BMC Plant Biol.

[17] Zhu A, Guo W, Gupta S, Fan W, Mower JP (2016). Evolutionary dynamics of the plastid inverted repeat: the effects of expansion, contraction, and loss on substitution rates. New Phytol.

[18] Vu H-T, Tran N, Nguyen T-D, Vu Q-L, Bui M-H, Le M-T (2020). Complete chloroplast genome of Paphiopedilum delenatii and phylogenetic relationships among Orchidaceae. Plants-Basel.

[19] Dong W, Xu C, Wu P, Cheng T, Yu J, Zhou S (2018). Resolving the systematic positions of enigmatic taxa: manipulating the chloroplast genome data of Saxifragales. Mol Phylogenet Evol.

[20] Li W, Liu Y, Yang Y, Xie X, Lu Y, Yang Z (2018). Interspecific chloroplast genome sequence diversity and genomic resources in Diospyros. BMC Plant Biol.

[21] Lee JH, Lee JW, Sung JS, Bang KH, Moon SG (2009). Molecular authentication of 21 Korean Artemisia species (Compositae) by polymerase chain reaction-restriction fragment length polymorphism based on trnL-F region of Chloroplast DNA. Biol Pharm Bull.

[22] Mahmood T, Hassan N, Nazar N, Naveed I (2011). Phylogenetic analysis of different Artemisia species based on Chloroplast Gene Rps11. Arch Biol Sci.

[23] Pellicer J, Valles J, Korobkov AA, Garnatje T (2011). Phylogenetic relationships of Artemisia subg. Dracunculus (Asteraceae) based on ribosomal and chloroplast DNA sequences. Taxon.

[24] Liu Y, Huo N, Dong L, Wang Y, Zhang S, Young HA (2013). Complete chloroplast genome sequences of Mongolia Medicine Artemisia frigida and phylogenetic relationships with other plants. PLoS ONE.

[25] Haghighi AR, Belduz AO, Vahed MM, Coskuncelebi K, Terzioglu S (2014). Phylogenetic relationships among Artemisia species based on nuclear ITS and chloroplast psba-trnh DNA markers. Biologia.

[26] Shen X, Wu M, Liao B, Liu Z, Bai R, Xiao S (2017). Complete chloroplast genome sequence and phylogenetic analysis of the Medicinal Plant Artemisia annua. Molecules.

[27] Kim G-B, Lim CE, Kim J-S, Kim K, Lee JH, Yu H-J et al. Comparative chloroplast genome analysis of Artemisia (Asteraceae) in East Asia: insights into evolutionary divergence and phylogenomic implications. BMC Genomics. 2020;21. 10.1186/s12864-020-06812-7 PMC7310033 32571207

[28] Huang R, Xie X, Chen A, Li F, Tian E, Chao Z (2021). The chloroplast genomes of four Bupleurum (Apiaceae) species endemic to Southwestern China, a diversity center of the genus, as well as their evolutionary implications and phylogenetic inferences. BMC Genomics.

[29] Jin J-J, Yu W-B, Yang J-B, Song Y, dePamphilis CW, Yi T-S (2020). GetOrganelle: a fast and versatile toolkit for accurate de novo assembly of organelle genomes. Genome Biol.

[30] Meng D, Xiaomei Z, Wenzhen K, Xu Z (2019). Detecting useful genetic markers and reconstructing the phylogeny of an important medicinal resource plant, Artemisia selengensis, based on chloroplast genomics. PLoS ONE.

[31] Qu X-J, Moore M, Li D-Z, Yi T (2019). PGA: a software package for rapid, accurate, and flexible batch annotation of plastomes. Plant Methods.

[32] Kearse M, Moir R, Wilson A, Stones-Havas S, Cheung M, Sturrock S (2012). Geneious Basic: an integrated and extendable desktop software platform for the organization and analysis of sequence data. Bioinformatics.

[33] Lehwark P, Greineri S (2019). GB2sequin-A file converter preparing custom GenBank files for database submission. Genomics.

[34] Dana A, Tuller T (2014). The effect of tRNA levels on decoding times of mRNA codons. Nucleic Acids Res.

[35] Menezes APA, Resende-Moreira LC, Buzatti RSO, Nazareno AG, Carlsen M, Lobo FP (2018). Chloroplast genomes of Byrsonima species (Malpighiaceae): comparative analysis and screening of high divergence sequences. Sci Rep.

[36] Abdullah MF, Shahzadi I, Waseem S, Mirza B, Ahmed I (2020). Chloroplast genome of Hibiscus rosa-sinensis (Malvaceae): comparative analyses and identification of mutational hotspots. Genomics.

[37] Frazer KA, Pachter L, Poliakov A, Rubin EM, Dubchak I (2004). VISTA: computational tools for comparative genomics. Nucleic Acids Res.

[38] Rozas J, Ferrer-Mata A, Sánchez-DelBarrio JC, Guirao-Rico S, Librado P, Ramos-Onsins SE, et al. Mol Biol Evol. 2017;34:3299–302. DnaSP 6: DNA Sequence Polymorphism Analysis of Large Data Sets. 10.1093/molbev/msx248 29029172

[39] Katoh K, Standley DM (2013). MAFFT multiple sequence alignment Software Version 7: improvements in performance and usability. Mol Biol Evol.

[40] Wang D, Zhang Y, Zhang Z, Zhu J, Yu J, Genomics (2010). Proteom Bioinf.

[41] Zhang D, Gao F, Jakovlić I, Zou H, Zhang J, Li WX (2020). PhyloSuite: an integrated and scalable desktop platform for streamlined molecular sequence data management and evolutionary phylogenetics studies. Mol Ecol Resour.

[42] Shaw J, Lickey EB, Schilling EE, Small RL (2007). Comparison of whole chloroplast genome sequences to choose noncoding regions for phylogenetic studies in angiosperms: the tortoise and the hare III. Am J Bot.

[43] Wu F-H, Chan M-T, Liao D-C, Hsu C-T, Lee Y-W, Daniell H (2010). Complete chloroplast genome of Oncidium Gower Ramsey and evaluation of molecular markers for identification and breeding in Oncidiinae. BMC Plant Biol.

[44] Nie X, Lv S, Zhang Y, Du X, Wang L, Biradar SS (2012). Complete chloroplast genome sequence of a major invasive species, Crofton Weed (Ageratina adenophora). PLoS ONE.

[45] Dong W, Xu C, Cheng T, Zhou S (2013). Complete chloroplast genome of Sedum sarmentosum and Chloroplast Genome Evolution in Saxifragales. PLoS ONE.

[46] Luo J, Hou B-W, Niu Z-T, Liu W, Xue Q-Y, Ding X-Y (2014). Comparative chloroplast genomes of photosynthetic orchids: insights into evolution of the Orchidaceae and Development of molecular markers for phylogenetic applications. PLoS ONE.

[47] Nazareno AG, Carlsen M, Lohmann LG (2015). Complete chloroplast genome of Tanaecium tetragonolobum: the First Bignoniaceae Plastome. PLoS ONE.

[48] McInerney JO (1998). GCUA: general codon usage analysis. Bioinformatics.

[49] Palmer JD, Thompson WF (1982). Chloroplast DNA rearrangements are more frequent when a large inverted repeat sequence is lost. Cell.

[50] Asaf S, Khan AL, Khan MA, Shahzad R, Lubna, Kang SM (2018). Complete chloroplast genome sequence and comparative analysis of loblolly pine (Pinus taeda L.) with related species. PLoS ONE.

[51] De las Rivas J, Lozano JJ, Ortiz AR (2002). Comparative analysis of chloroplast genomes: functional annotation, genome-based phylogeny, and deduced evolutionary patterns. Genome Res.

[52] Song W, Chen Z, Shi W, Han W, Feng Q, Shi C (2022). Comparative Analysis of Complete Chloroplast Genomes of Nine Species of Litsea (Lauraceae): hypervariable regions, positive selection, and phylogenetic relationships. Genes.

[53] Wang R-J, Cheng C-L, Chang C-C, Wu C-L, Su T-M, Chaw S-M (2008). Dynamics and evolution of the inverted repeat-large single copy junctions in the chloroplast genomes of monocots. BMC Evol Biol.

[54] Lee YS, Park JY, Kim J-K, Lee HO, Park H-S, Lee S-C (2016). The complete chloroplast genome sequences of Artemisia gmelinii and Artemisia capillaris (Asteraceae). Mitochondrial DNA Part B.

[55] Curci PL, Sonnante G (2016). The complete chloroplast genome of Cynara Humilis. Mitochondrial DNA Part A.

[56] Zhang J, Jiang Z, Su H, Zhao H, Cai J (2019). The complete chloroplast genome sequence of the endangered species Syringa pinnatifolia (Oleaceae). Nord J Bot.

[57] Quax TEF, Claassens NJ, Soell D, van der Oost J (2015). Codon Bias as a Means to Fine-Tune Gene expression. Mol Cell.

[58] Iram S, Hayat MQ, Tahir M, Gul A, Abdullah, Ahmed I (2019). Chloroplast Genome sequence of Artemisia scoparia: comparative analyses and screening of mutational hotspots. Plants (Basel).

[59] Miao H, Bao J, Li X, Ding Z, Tian X (2022). Comparative analyses of chloroplast genomes in Red Fuji apples: low rate of chloroplast genome mutations. PeerJ.

[60] Pei J, Wang Y, Zhuo J, Gao H, Vasupalli N, Hou D (2022). Complete chloroplast genome features of Dendrocalamusfarinosus and its comparison and evolutionary analysis with other Bambusoideae Species. Genes.

[61] Henriquez CL, Abdullah, Ahmed I, Carlsen MM, Zuluaga A, Croat TB (2020). Molecular evolution of chloroplast genomes in Monsteroideae (Araceae). Planta.

[62] Bausher MG, Singh ND, Lee S-B, Jansen RK, Daniell H (2006). The complete chloroplast genome sequence of Citrus sinensis (L.) Osbeck var Ridge Pineapple: organization and phylogenetic relationships to other angiosperms. BMC Plant Biol.

[63] Morgante M, Hanafey M, Powell W (2002). Microsatellites are preferentially associated with nonrepetitive DNA in plant genomes. Nat Genet.

[64] Dong W, Xu C, Cheng T, Lin K, Zhou S (2013). Sequencing Angiosperm Plastid genomes made Easy: A Complete Set of Universal primers and a case study on the phylogeny of Saxifragales. Genome Biol Evol.

[65] Kuang D-Y, Wu H, Wang Y-L, Gao L-M, Zhang S-Z, Lu L (2011). Complete chloroplast genome sequence of Magnolia kwangsiensis (Magnoliaceae): implication for DNA barcoding and population genetics. Genome.

[66] Wang Y, Wang S, Liu Y, Yuan Q, Sun J, Guo L (2021). Chloroplast genome variation and phylogenetic relationships of atractylodes species. BMC Genomics.

[67] Li X, Yang Y, Henry RJ, Rossetto M, Wang Y, Chen S (2015). Plant DNA barcoding: from gene to genome. Biol Rev.

[68] Jiang D-H (2024). The complete chloroplast genome sequence of *Artemisia stechmanniana* (*Asteraceae*): genome structure and phylogenetic analysis. Biologia.

[69] Jin G, Li W, Song F (2023). Comparative analysis of complete *Artemisia* subgenus *Seriphidium* (Asteraceae: Anthemideae) chloroplast genomes: insights into structural divergence and phylogenetic relationships. BMC Plant Biol.

[70] Shahzadi I, Abdullah, Mehmood F, Ali Z, Ahmed I, Mirza B (2020). Chloroplast genome sequences of Artemisia maritima and Artemisia absinthium: comparative analyses, mutational hotspots in genus Artemisia and phylogeny in family Asteraceae. Genomics.

