# Supplementary material for: Comparative and phylogenetic analysis of the complete chloroplast genomes of 10 Artemisia selengensis resources based on high-throughput sequencing
Source: BMC Genomics. 2024 Jun 5;25:561. doi: 10.1186/s12864-024-10455-3 (PMC11151499; doi:10.1186/s12864-024-10455-3)
Supplement: Supplementary file 1 — Supplementary Material 1 [file 12864_2024_10455_MOESM1_ESM.docx]

**Supplementary Table1.**

Summary of sequencing data quality

| **Sample** | **Raw Base(G)** | **Clean Base(G)** | **Effective Rate(%)** | **Error Rate(%)** | **Q20(%)** | **Q30(%)** | **GC Content(%)** |
| --- | --- | --- | --- | --- | --- | --- | --- |
| JN1 | 5.49 | 5.45 | 99.33 | 0.03 | 97.67 | 93.25 | 35.78 |
| HWB | 5.53 | 5.5 | 99.45 | 0.03 | 97.47 | 92.71 | 36.28 |
| AC | 5.9 | 5.86 | 99.39 | 0.03 | 97.32 | 92.4 | 36.16 |
| YN | 5.66 | 5.62 | 99.37 | 0.03 | 97.28 | 92.31 | 37.36 |
| HQ | 5.78 | 5.74 | 99.43 | 0.03 | 97.49 | 92.76 | 36.41 |
| HY | 5.63 | 5.6 | 99.44 | 0.03 | 97.27 | 92.26 | 36.20 |
| JN2 | 5.64 | 5.61 | 99.39 | 0.03 | 97.34 | 92.45 | 37.00 |
| HC | 5.64 | 5.61 | 99.45 | 0.03 | 97.27 | 92.28 | 36.11 |
| HWS | 5.64 | 5.61 | 99.42 | 0.03 | 97.41 | 92.62 | 36.17 |
| JS | 5.59 | 5.55 | 99.41 | 0.03 | 96.46 | 90.5 | 36.09 |
