# Supplementary material for: Comparative and phylogenetic analysis of the complete chloroplast genomes of 10 Artemisia selengensis resources based on high-throughput sequencing
Source: BMC Genomics. 2024 Jun 5;25:561. doi: 10.1186/s12864-024-10455-3 (PMC11151499; doi:10.1186/s12864-024-10455-3)
Supplement: Supplementary file 2 — Supplementary Material 2 [file 12864_2024_10455_MOESM2_ESM.docx]

**Supplementary Table2.**

List of the Chloroplast genomes used for the phylogenetic analysis

| **NO.** | **Name** | **GenBank numbers** |
| --- | --- | --- |
| 1 | *Artemisia annua* | NC_034683 |
| 2 | *Artemisia argyi* | NC_030785 |
| 3 | *Artemisia capillaris* | NC_031400 |
| 4 | *Artemisia desertorum* | NC_063905 |
| 5 | *Artemisia freyniana f. discolor* | NC_049570 |
| 6 | *Artemisia frigida* | NC_020607 |
| 7 | *Artemisia fukudo* | NC_044156 |
| 8 | *Artemisia gmelinii* | NC_031399 |
| 9 | *Artemisia hallaisanensis* | NC_049571 |
| 10 | *Artemisia maritima* | NC_045093 |
| 11 | *Artemisia montana* | NC_025910 |
| 12 | *Artemisia ordosica* | NC_046571 |
| 13 | *Artemisia scoparia* | NC_045286 |
| 14 | *Artemisia selengensis* | NC_039647 |
| 15 | *Artemisia stolonifera* | NC_049572 |
| 16 | *Chrysanthemum indicum* | NC_020320 |
| 17 | *Chrysanthemum x morifolium* | NC_020092 |
| 18 | *Guizotia abyssinica* | NC_010601 |
| 19 | *Helianthus annuus* | NC_007977 |
| 20 | *Mikania micrantha* | NC_031833 |
| 21  22  23  24  25  26  27 | *Helianthus argophyllus*  *Chrysanthemum boreale*  *Helianthus atrorubens*  *Cynara humilis*  *Cynara baetica*  *Cynara cornigera*  *Helianthus debilis* | NC_030275  NC_037388  NC_058796  NC_027113  NC_028005  NC_028006  NC_030173 |
